# Pain and Sedative Medication Use Among Individuals With Inflammatory Bowel Disease: A Nationwide Population‐Based Cohort Study

**DOI:** 10.1111/apt.70247

**Published:** 2025-07-02

**Authors:** Samantha Baillie, Sonia Saxena, Nishani Jayasooriya, Alex Bottle, Irene Petersen, Jonathan Blackwell, Richard Pollok

**Affiliations:** ^1^ Institute for Infection and Immunity City St George's University of London London UK; ^2^ School of Public Health Imperial College London London UK; ^3^ Research Department of Primary Care and Population Health University College London London UK; ^4^ Edinburgh Inflammatory Bowel Disease Unit Western General Hospital Edinburgh UK; ^5^ Department of Gastroenterology St George's University Hospitals NHS Foundation Trust London UK

**Keywords:** abdominal pain, Crohn's disease, epidemiology, general practice, guidelines, inflammatory bowel disease, ulcerative colitis

## Abstract

**Background:**

Individuals with inflammatory bowel disease (IBD) often experience pain, mood disturbances, and sleep disruption, which may lead to greater use of pain‐relieving and sedative medications compared with the general population. These are associated with increased mortality, paradoxical worsening of pain, and inappropriate IBD treatment discontinuation. Chronic prescribing and co‐prescribing increase the risk of respiratory depression, dependence, and overdose.

**Methods:**

Using Clinical Practice Research Datalink, a large nationally representative dataset, we examined the annual prevalence of total, chronic (> 90 days opioids; > 28 days sedatives), and co‐prescribed opioids, gabapentinoids and sedatives in adults with incident IBD from January 2010 to December 2019. Multivariable regression identified predictors of chronic or co‐prescribing.

**Results:**

Among 17,388 individuals, over 20% were prescribed a pain or sedative medication each year. Annual prevalence for opioids and gabapentinoids increased (13.6%–14% and 2.5%–5.6%, respectively) while sedative prevalence remained stable (8.4%). Chronic prescribing increased for strong opioids (3.6%–4.6%), weak opioids (3.6%–3.7%) and sedatives (4.2%–4.4%). Between 4.2% and 6.9% of individuals per year were co‐prescribed opioids, gabapentinoids, and/or sedatives. Female sex, smoking, older age at diagnosis, Crohn's disease, and a diagnosis of inflammatory arthropathy, irritable bowel syndrome, fibromyalgia, or anxiety/depression were significantly associated with chronic and/or co‐prescriptions of opioids or sedatives.

**Conclusion:**

A substantial proportion of individuals with IBD are prescribed pain and sedative medications, including long‐term and co‐prescriptions. Identifying high‐risk patients is essential to ensure they are prioritised for limited resources, such as psychological therapies, as alternatives to harmful prescriptions.

## Graphical abstract

Over 20% of individuals with IBD were prescribed pain or sedative medications annually, with rising chronic and co‐prescribing rates. Risk factors included female sex, older age, Crohn's disease, and mental health conditions. Targeted interventions are needed to reduce harmful prescribing and promote safer alternatives.

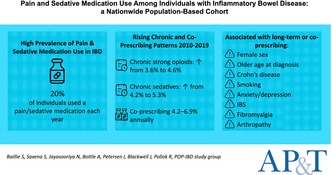

## 1 Introduction

Concerns about the harm associated with long‐term and overlapping prescribing of pain and sedative medications have led to guidelines warning against these practices [1, 2, 3, 4]. Despite this, prescribing rates in the general population continue to rise [5, 6, 7]. However, contemporary data specific to the inflammatory bowel disease (IBD) population on chronic and co‐prescribing of opioids, gabapentinoids, benzodiazepines, and Z‐drugs (non‐benzodiazepine hypnotics acting at the benzodiazepine receptor) are lacking.

Individuals with IBD frequently experience chronic pain, poor sleep, and mood disorders, with or without active disease, making them likely to use pain and sedative medications [8, 9, 10, 11, 12, 13]. Sleep disturbances are common due to nocturnal bowel movements, pain, corticosteroid use, and other poorly understood mechanisms, which may drive patients toward sleep aids such as benzodiazepines or Z‐drugs [14, 15]. Additionally, IBD‐related chronic pain can arise from the disease itself or associated conditions such as inflammatory arthropathies and oral ulcers, as well as from visceral hypersensitivity and comorbid irritable bowel syndrome (IBS) [16]. Pregabalin and gabapentin are not approved for these conditions; however, more than half of gabapentinoid prescriptions are for off‐label indications [17]. Surgery, which remains a frequent necessity for IBD patients, is a known risk factor for long‐term pain medication use, especially opioids [18, 19]. Furthermore, depression and anxiety are more prevalent in IBD, increasing the likelihood of prolonged and potentially harmful use of medications such as benzodiazepines [13, 20]. Managing these symptoms pharmacologically is challenging, as these medications are associated with serious side effects, including opioid‐induced hyperalgesia, respiratory depression, and overdose [21, 22, 23]. Strong opioids (e.g., morphine and oxycodone) provide potent pain relief for severe pain but carry a high risk of dependence, respiratory depression, and overdose. Weak opioids (e.g., codeine and tramadol) are less potent but still pose risks of addiction, side effects, and potential for misuse, especially with prolonged use. The risks increase further when multiple pain or sedative medications are co‐prescribed, leading to a higher likelihood of hospitalisation, infection, and death [22, 24, 25]. Chronic prescribing (> 90 days for opioids, > 28 days for sedatives) carries additional concerns, including dependence, paradoxically worsened pain and sleep, and increased mortality [22, 26, 27]. In IBD‐specific studies, opioid use did not improve pain scores [28], abdominal pain, or quality of life [21] and the use of pain and sedative medications has been linked to higher rates of hospitalisation, emergency visits, serious infections, inappropriate biologic discontinuation, and increased mortality [29, 30, 31, 32].

In North America, individuals with IBD are more likely to be prescribed opioids, yet few studies have examined opioid use in European IBD populations [31, 33]. Similarly, benzodiazepines (e.g., diazepam and alprazolam) and Z‐drugs (e.g., zopiclone and zolpidem) are more frequently prescribed to individuals with IBD [24, 29], while global research on their use in this context remains limited [31, 33]. Gabapentinoids may have analgesic effects that reduce the need for opioids, either by replacing them or being used in conjunction, and it has been suggested that they have anti‐inflammatory properties, reducing mucosal inflammation [34]. However, concerns have emerged about increasing reports of misuse, dependence, and their detection in post‐mortem toxicology analyses [6, 35, 36, 37]. Data on prescribing prevalence, co‐prescribing practices, and overall use of gabapentinoids in IBD remain lacking.

Given the paucity of data regarding trends in pain and sedative medication use in the IBD population, particularly gabapentinoids, and concerns about their associated harms, we aimed to evaluate their use in a nationally representative UK cohort. Furthermore, we also aimed to determine risk factors associated with co‐prescribed or chronically prescribed pain and sedative medications to identify individuals at most risk of harm.

## 2 Methods

### 2.1 Data Source

Using the Clinical Practice Research Datalink‐GOLD (CPRD), we identified a retrospective cohort of individuals who were over 18 years of age and had an incident diagnosis of IBD during the period from January 1, 2010, to December 31, 2019. CPRD is a UK‐based epidemiological research resource that provides anonymised, real‐world data from primary care. It includes longitudinal, detailed, patient‐level medical records from millions of patients, including information on diagnoses, treatments, test results, prescriptions, and referrals, with a median follow‐up of 9.4 years during our study period. The database covers 6.9% of the population and is broadly representative of the UK population in terms of age, sex and ethnicity [38]. Participating practices are required to remain ‘up to standard’ (UTS) with a high level of accuracy and completeness to continue contributing to the dataset. The CPRD dataset was linked to the Hospital Episode Statistics (HES) database to obtain data on all admissions and operations in National Health Service hospitals in England. Ethical approval was granted by the Health Research Authority Research Ethics Committee (ERAP protocol number 20_000248).

### 2.2 Cohort Construction

The retrospective incident IBD cohort included all individuals over the age of 18 with their first diagnostic Read code for CD or UC at least 1 year after registering with a UTS GP practice. We excluded individuals who had read codes for both CD and UC, or indeterminate codes, following previously validated methods [39]. Patients were followed up until the study endpoint, patient death, patient transfer out of the GP practice or the GP practice ceased contributing data to CPRD. Patient data was extracted at the time of IBD diagnosis for age, sex, IBD type, body mass index (BMI), smoking status, index of multiple deprivation (IMD), region and comorbidities including anxiety or depression, fibromyalgia, IBS and inflammatory arthropathy. Age at the time of diagnosis was the number of years between the recorded year of birth and the IBD diagnosis date [40]. The IBD type was Crohn's disease (CD) or ulcerative colitis (UC) according to the diagnostic CPRD medcode. The BMI was the most recent value within the past 3 years that was recorded directly or could be calculated from a recorded height and weight. Smoking status was categorised at the time of diagnosis as “never smoker,” “previous smoker” or “current smoker” based on codes for smoking status in the 2 years before the incident IBD diagnosis [41]. CPRD smoking status is accurate to within 1% of self‐reported smoking habits in national surveys [42]. The IMD is a measure used in the UK to assess the relative deprivation of areas based on a combination of factors, including income, employment, education, health, crime, housing, and living environment. A scoring system is used to arrange areas into quintiles, with one being the least deprived and five being the most deprived [43]. The recording of IMD was limited by factors, including its availability in CPRD only for individuals registered with a GP in England and was recorded for 8173 (52.5%) individuals in 2010 to 2076 (15.9%) in 2019. Geographical regions 1–10 represent the English Strategic Health Authorities and regions 11–13 represent Wales, Scotland and Northern Ireland, respectively. Individuals with anxiety and/or depression symptoms or diagnoses before IBD diagnosis were identified using medcodes for depression diagnoses, depressive symptoms, anxiety disorder diagnoses, or anxiety symptoms. Medcodes were used to identify those with IBS, inflammatory arthropathy, and fibromyalgia diagnoses before the index IBD diagnosis date.

### 2.3 Outcomes

#### 2.3.1 Annual Prevalence of Pain and Sedative Medication Use in the IBD Population

We calculated the number of individuals prescribed strong opioids, weak opioids, gabapentinoids, benzodiazepines, and Z‐drugs per 100 patients each year among our cohort in 2010–2019. All primary care prescriptions in CPRD, including the drug name and date of prescription, are recorded automatically. Prescription episodes for opioids, gabapentinoids, benzodiazepines, and Z‐drugs were identified using relevant prodcodes (see Data S1). Prescriptions were included only if they commenced at least 1 year after a practice was UTS and if they were prescribed to an individual over the age of 18 with a diagnosis of IBD.

Opioids were sub‐categorised into weak (codeine, dihydrocodeine, dextropropoxyphene, and tramadol) and strong (all other) opioids (see Data S1 for complete list). Opioid‐containing medications were stratified according to the opioid product (e.g., co‐codamol contains codeine and is a weak opioid). Z‐drugs (non‐benzodiazepine sedatives acting on the benzodiazepine receptor) were zolpidem, zopiclone, and zaleplon.

### 2.4 Chronic Prescriptions

A “chronic” prescription was defined as a continuous prescription lasting more than 90 days for opioids or 28 days for sedatives (Z‐drugs and benzodiazepines), in line with national guidance [1, 4, 44]. A preliminary analysis of prescriptions indicated that the median prescription duration was 28 days for all our medications of interest so an episode of continuous prescription was defined as ending when there was a gap of ≥ 35 days between prescriptions (allowing for prescription duration 28 days plus 1 week to obtain a repeat prescription) or the individual reached the end of their follow‐up period (December 31st 2019, patient death, patient transferred out of the practice or the GP practice no longer contributes data to CPRD).

Prolonged use of gabapentinoids requires periodic re‐evaluation due to dependency and side effects. However, no maximum period is recommended, and therefore, chronic gabapentinoid use was not evaluated.

### 2.5 Co‐Prescription

Prescriptions for opioids, gabapentinoids, benzodiazepines, and/or Z‐drugs were considered ‘co‐prescribed’ if their durations overlapped.

### 2.6 Risk Factors and Covariates

We reviewed the literature and identified a list of hypothesised risk factors present at the time of diagnosis that may increase the risk of an individual receiving a chronic or co‐prescription for a pain medication or sedative during the follow‐up period [24, 45, 46, 47, 48, 49]. These included sex, age at diagnosis, IBD type, smoking status, and comorbidities including anxiety and/or depression, IBS, inflammatory arthropathy, and fibromyalgia (see above for definitions).

Those undergoing IBD‐related surgery during follow‐up were identified using relevant OPCS codes recorded in HES‐linked records. Surgery is associated with both pain and anxiety/depression [31, 50]; therefore, undergoing surgery during the follow‐up period was adjusted for in the regression analysis. Similarly, chronic and/or refractory steroid use was considered a proxy indicator of disease activity, which may be linked to increased pain, and as such was included as an adjustment factor in the analysis. Chronic steroid prescriptions were those continuing longer than 56 days and refractory to more than two courses within 1 year, in line with international guidelines [2, 51, 52] Steroid prescriptions were included only if issued within 1 month of a documented gastrointestinal symptom, to establish relation to IBD disease activity. To assess the impact of potentially incomplete coding of gastrointestinal symptoms, a sensitivity analysis was conducted that included all steroid prescriptions during the follow‐up period, regardless of their relation to gastrointestinal symptoms.

### 2.7 Statistical Analysis

We calculated the annual prevalence of strong opioid, weak opioid, gabapentinoid, benzodiazepine, and Z‐drug prescribing among our cohort for the years 2010–2019. The population denominator was the total number of individuals who were over the age of 18 at the start of the year, with a previous incident diagnosis of IBD, and registered with a UTS practice for the duration of the calendar year. The numerator was the total number of these individuals who were prescribed the drug of interest, e.g., a gabapentinoid, in the given calendar year.

We calculated the annual prevalence of chronic prescribing for strong opioids, weak opioids, benzodiazepines, and Z‐drugs, including only individuals with prescriptions lasting longer than 28 days (for sedatives) or 90 days (for opioids) in the numerator. The annual prevalence of co‐prescribing was determined by including all individuals who received overlapping prescriptions during the specified year in the numerator.

We used a multiple logistic regression model to identify potential predictors of an increased likelihood of receiving a chronic or co‐prescription in the 5 years following IBD diagnosis. Factors were selected for inclusion in the model through a directed acyclic graph (DAG), which was constructed to represent the hypothesised causal relationships between exposures and the outcome. (Supporting Information Figure S1).

The variables in this model included sex, age at diagnosis, IBD type, smoking status, and comorbidities including anxiety and/or depression, IBS, inflammatory arthropathy, fibromyalgia, chronic/refractory steroid use, and IBD‐related surgery. Firth's penalised logistic regression was used to reduce bias and prevent separation issues caused by rare events in certain predictor groups.

Data were analysed using STATA 18 (Statacorp LP, College Station, TX, USA) and R version 4.4.0 (http://www.r‐project.org).

## 3 Results

### 3.1 Patient Characteristics

The annual number of patients meeting the inclusion criteria for the annual prescribing trends cohort ranged from 13,086 in 2019 to 17,388 in 2013. While there were temporal changes in the proportion of missing data, the overall distribution of patient characteristics, including sex (49.8%–50.5% female), median age (52–53), and the proportion of individuals with CD (30.4%–33.1%), remained consistent between 2010 and 2019. An increasing proportion of patients resided in the devolved nations during the latter years of the study (35.8% in 2013 to 78.5% in 2019) and there was a rise in the proportion of individuals with anxiety/depression (23.1%–34.1%) and fibromyalgia (1.1%–2.2%) diagnoses, accompanied by a slight decline in the proportion of individuals with IBS (11.9%–7.2%) (Table S1).

There were 4901 individuals with at least 5 years' follow‐up who met the inclusion criteria for the regression model over the decade: 49% were female, 32% had CD and the median age was 47 (Table 1).

**TABLE 1:** Baseline characteristics for adult individuals with an incident inflammatory bowel disease diagnosis after 1st January 2010 with at least 5 years of follow‐up.

	Individuals (*n* = 4901)	
Age		
Median, (25th–75th percentile)	47	(33–62)
Sex		
Female	2414	(49%)
IBD type		
Crohn's disease	1578	(32%)
Ulcerative colitis	3323	(68%)
Smoking status^a^		
Current	771	(16%)
Previous	1385	(28%)
Never	1582	(32%)
Missing data	1163	(24%)
Comorbidities^b^		
Anxiety or depression	1200	(24%)
Irritable bowel syndrome	676	(14%)
Inflammatory arthropathy	129	(3%)
Fibromyalgia	53	(1%)
Steroid use^c^	765	(16%)
Surgery^d^	147	(3%)

Abbreviation: IBD, inflammatory bowel disease.

^a^ Smoking status: most recent status recorded in the previous 2 years.

^b^ Comorbidities: individuals with a previous diagnosis of each relevant comorbidity at the time of inflammatory bowel disease diagnosis.

^c^ Chronic and/or refractory steroid use is defined as a steroid prescription lasting more than 56 days and/or refractory use is defined as more than one steroid prescription within a one‐year period within 30 days of a recorded gastrointestinal symptom.

^d^ IBD‐related surgery in the 5 year follow‐up period.

### 3.2 Annual Trends

**FIGURE 1:**
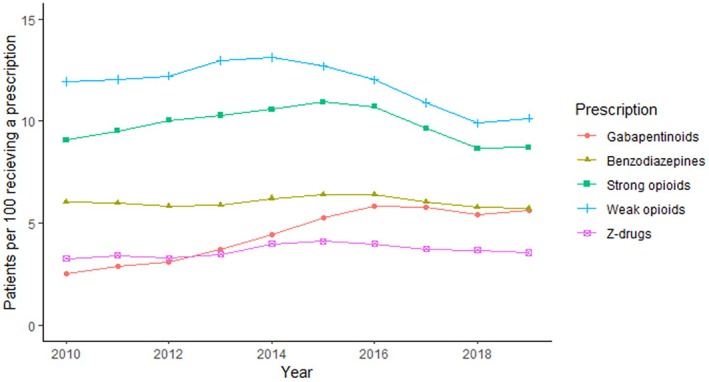
Annual trends in the prescribing of pain and sedative medications among individuals with a diagnosis of inflammatory bowel disease in the years 2010–2019.

While strong opioid, weak opioid and benzodiazepine prevalence decreased during the study period, Z‐drug and gabapentinoid prevalence increased (Figure 1, Table 2).

**TABLE 2:** Annual prevalence of prescriptions in the years 2010–2019.

Year	Annual prevalence (patients per 100) of prescriptions										
	Any drug		Strong opioids		Weak opioids		Benzodiazepines		Z‐drugs		Gabapentinoids
	Total	Chronic	Total	Chronic^a^	Total	Chronic	Total	Chronic^b^	Total	Chronic^b^	Total
2019	20.2	8.2	8.7	4.6	10.1	3.7	5.8	2.8	3.6	2.2	5.6
2018	20.7	8.6	8.7	4.5	9.9	3.7	5.8	2.8	3.7	2.0	5.4
2017	20.8	8.6	9.6	4.9	10.9	3.9	6.1	2.9	3.8	2.2	5.8
2016	21.7	8.8	10.7	4.9	12.0	4.3	6.4	3.2	4.0	2.2	5.8
2015	23.0	9.6	10.9	5.2	12.7	4.3	6.4	3.3	4.1	2.2	5.3
2014	23.9	9.7	10.6	5.0	13.1	4.4	6.2	3.4	4.0	2.3	4.5
2013	23.8	9.6	10.3	4.7	13.0	4.0	5.9	3.0	3.5	2.0	3.7
2012	22.3	9.2	10.0	4.4	12.2	4.0	5.8	2.9	3.3	2.0	3.1
2011	20.7	8.9	9.5	4.1	12.0	3.9	6.0	3.2	3.4	2.1	2.9
2010	21.1	9.0	9.1	3.6	11.9	3.6	6.0	3.3	3.3	2.1	2.5

^a^ Chronic opioid prescription: more than 90 days of continuous prescription.

^b^ Chronic benzodiazepine or Z‐drug prescription: more than 28 days of continuous prescription.

Between 8.2% and 9.7% of individuals had a chronic prescription depending on the calendar year studied (Table 2). Chronic prescribing of all drugs except benzodiazepines increased over the study period: strong opioids from 3.6% to 4.6%, weak opioids 3.6% to 3.7%, Z‐drugs 2.1% to 2.2%, and benzodiazepines 3.3% to 2.8%.

Co‐prescribing of two or more pain/sedative medications increased over the decade. Between 3.9% and 6.3% of all patients were co‐prescribed opioids with gabapentinoid or sedative medication, whereas 3.0%–3.4% of individuals were co‐prescribed sedatives with an opioid or gabapentinoid, and 1.4%–3.2% of individuals were co‐prescribed a gabapentinoid with an opioid or sedative (Table S3). In 2019, the proportion of co‐prescriptions was highest for gabapentinoids (56% of all gabapentinoid prescriptions) compared with 35% of opioid prescriptions and 39% of sedative prescriptions (Table 3).

**TABLE 3:** Co‐prescribed medications in the year 2019.

	Primary medication		
	Opioid	Gabapentinoid	Sedative
Total co‐prescriptions	621	411	427
Total prescriptions	1776	737	1105
(Co‐prescriptions/Total prescriptions) x 100	35%	56%	39%
Co‐prescribed medication			
Opioid			
Co‐prescriptions		350	401
(Co‐prescriptions/Total prescriptions) x 100		47%	36%
Gabapentinoid			
Co‐prescriptions	350		117
(Co‐prescriptions/Total prescriptions) x 100	20%		11%
Sedative			
Co‐prescriptions	401	117	
(Co‐prescriptions/Total prescriptions) x 100	23%	16%	

*Note:* Total and % of opioid, gabapentinoid and sedative prescriptions that were co‐prescribed in the year 2019, and the secondary medication that they were prescribed with.

### 3.3 Multivariable Regression Analysis

Factors significantly associated with all prescribing patterns of interest (chronic strong opioid, chronic sedative, or co‐prescribing) were older age at diagnosis, female sex, current smoking, anxiety/depression, IBS, and fibromyalgia prior to IBD diagnosis (Table 4). Inflammatory arthropathy was associated with chronic strong opioid prescribing and co‐prescribing but not chronic sedative prescribing. CD was associated only with increased risk of chronic strong opioid prescribing.

**TABLE 4:** Multivariable regression analysis of risk factors associated with prescribing patterns of interest; chronic prescribing of strong opioids (> 90 days), chronic prescribing of sedatives (> 28 days) or co‐prescribing more than 1 drug of interest.

Predictor		Chronic strong opioid prescription			Chronic sedative prescription			Co‐prescription		
		OR	95% confidence interval	*p*	OR	95% confidence interval	*p*	OR	95% confidence interval	*p*
Age at diagnosis	—	1.02	1.01–1.03	< 0.001	1.02	1.02–1.03	< 0.001	1.02	1.01–1.03	< 0.001
Sex	Female	1.79	1.42–2.26	< 0.001	1.54	1.24–1.90	< 0.001	1.83	1.47–2.29	< 0.001
IBD type^a^	Ulcerative colitis	1.00	—	—	1.00	—	—	1.00	—	—
	Crohn's disease	1.52	1.17–1.97	0.002	0.89	0.69–1.14	0.357	1.11	0.86–1.42	0.412
Smoking status^b^	Never smoker	1.00	—	—	1.00	—	—	1.00	—	—
	Previous smoker	1.05	0.77–1.43	0.762	1.29	0.98–1.71	0.071	1.20	0.90–1.60	0.204
	Current smoker	2.17	1.60–2.94	< 0.001	2.30	1.73–3.06	< 0.001	2.27	1.70–3.02	< 0.001
Comorbidity	Anxiety or depression	2.49	1.90–3.25	< 0.001	3.11	2.44–3.97	< 0.001	2.87	2.24–3.67	< 0.001
	Irritable bowel syndrome	1.64	1.19–2.23	0.003	1.45	1.07–1.96	0.018	1.66	1.23–2.21	0.001
	Inflammatory arthropathy	3.39	2.03–5.46	< 0.001	1.01	0.49–1.85	0.984	1.99	1.13–3.32	0.018
	Fibromyalgia	8.98	4.87–16.55	< 0.001	3.73	1.95–6.90	< 0.001	5.41	2.93–9.91	< 0.001
Steroids^c^	—	1.85	1.06–3.04	0.032	1.09	0.57–1.88	0.787	1.57	1.21–2.03	0.001
Surgery^d^	—	1.56	1.17–2.05	0.002	1.66	1.29–2.13	< 0.001	1.80	1.06–2.89	0.031

Abbreviations: IBD, inflammatory bowel disease; OR, odds ratio.

^a^ Reference group is ulcerative colitis.

^b^ Reference smoking status is “never smoker.”

^c^ Chronic or refractory steroid use during follow up as a proxy for disease activity. Chronic use defined as steroid prescriptions lasting more than 56 days and refractory use defined as more than one steroid prescription within a 1‐year period.

^d^ Reference group is no surgery during follow‐up.

### 3.4 Key Findings

Each year, one in five individuals with IBD received a prescription for an opioid, gabapentinoid, or sedative, with nearly half classified as chronic. This is important because chronic use of these medications has previously been associated with dependence, pain and increased mortality [22, 26, 27]. Gabapentinoid use more than doubled in the UK IBD population from 2.5% to 5.6% between 2010 and 2019. Despite a decade of growing concerns about opioid‐related mortality, dependence, and increased pain sensitivity, which led to guidelines discouraging first‐line and long‐term use, more than one in 10 patients were still prescribed opioids in 2019. Additionally, 5.9% and 4.2% of the population continued to receive chronic prescriptions for opioids and sedative medications, respectively, in 2019 (Table S2).

Current smokers were more than twice as likely to receive chronic prescriptions for strong opioids or sedatives and also to have a co‐prescription of pain or sedative medications relative to non‐smokers. Female sex, older age at IBD diagnosis, and comorbid depression, anxiety, IBS, fibromyalgia, or inflammatory arthropathy were also significantly associated with receiving a chronic or co‐prescription.

Individuals with a diagnosis of CD were one and a half times more likely to be prescribed strong opioids long‐term compared with individuals with UC.

### 3.5 Findings in Context

The findings of this study add to the limited data available on prescribing patterns for pain and sedative medications in IBD populations in the UK, which differ from trends observed in the United States. Previous research by Burr et al. [31] demonstrated a rising trend in opioid use in the IBD population during the late 1990s and early 2000s. However, our findings suggest that opioid prescribing has since plateaued, with an annual prevalence of about 10%, contrasting with US data indicating that 21% of IBD outpatients are opioid users, against the backdrop of the wider opioid epidemic [53]. Even so, opioid use in the UK IBD population has not reduced, as might be expected with the increasing number of licensed therapies for the management of IBD [54]. Data from Public Health England found that 13% of the general population received an opioid prescription in 2017/18, which is similar to the 14% seen in our IBD cohort in 2018. However, individuals with IBD have higher prescribing rates for gabapentinoids (5.4% vs. 3.3%), benzodiazepines (5.8% vs. 3.1%), and Z‐drugs (3.7% vs. 2.3%) [55]. Co‐prescribing patterns in this study were similar to those in the general population as reported in a Scottish study, in which 49% of gabapentinoids were co‐prescribed with opioids and 27% with benzodiazepines. The overall rate of gabapentinoid use was lower in the Scottish study (3.7% of the population) compared with 5.4% shown in this study. Alarmingly, the Scottish study also reported an increase in drug‐related deaths alongside the rise in gabapentinoid prescriptions [56]. To our knowledge, our study is the first to examine gabapentinoid prescribing in IBD patients and reveals a significant increase in their use. Although there are no specific guidelines addressing gabapentinoid use in IBD, existing literature raises concerns about their potential harms, including dependency, anxiety and suicidal ideation, severe exacerbations of COPD, and increased rates of drug‐related deaths, particularly with long‐term use or when co‐prescribed with other medications [56, 57, 58, 59, 60]. This trend is concerning given the lack of robust evidence supporting the efficacy of gabapentinoids in managing IBD‐related pain [16, 61].

Data from North America demonstrate higher sedative use in IBD populations compared with non‐IBD controls, but comparable data from Europe are lacking. Our study demonstrated 8.4% of the IBD population received a sedative prescription in 2019, with over half being chronic prescriptions (Table S4) and 39% co‐prescribed with either an opioid or gabapentinoid medication, which increases the risk of drug‐related harm.

Our study demonstrated that women and those diagnosed with IBD at older ages were at increased risk of chronic sedative and strong opioid prescriptions, as well as co‐prescriptions of these medications. This is particularly worrisome as one US study found that hospitalisations due to poisoning from prescribed opioids and sedatives were more common in women and those over the age of 34 [62].

In our study population, 24% had comorbid depression or anxiety, conditions which are known to be associated with both IBD and opioid prescriptions [13, 20, 63]. In keeping with previous studies, our study found that individuals with depression or anxiety were more than twice as likely to receive a chronic strong opioid prescription. Opioid use poses a particular risk to individuals with psychiatric comorbidity as it can also exacerbate depression; thus, opioid stewardship is especially important within the IBD population [64].

Chronic opioid use has increased slightly despite a modest decline in overall prescribing, suggesting a shift toward more selective use among individuals with complex or persistent symptoms. Heightened awareness of opioid‐related harms has likely reduced new initiations, yet those with refractory pain or limited access to non‐pharmacological alternatives may remain on long‐term regimens. Benzodiazepine prescribing, both total and chronic, has declined slightly, likely reflecting growing recognition of risks including dependence, cognitive impairment, and stricter prescribing guidelines. In contrast, Z‐drug use has risen moderately, with only a slight increase in chronic use, suggesting increased prescribing for sleep disturbances in IBD, though their lower risk of tolerance and dependence may reduce chronic use. These patterns highlight the influence that regulatory changes and clinician education can have, suggesting the potential for similar strategies to drive safer prescribing practices in the future.

### 3.6 Strengths and Limitations

To our knowledge, this is the first population‐based study to examine the prescribing of gabapentinoid and sedative medication in IBD within Europe and the only recent update to the limited literature looking at opioid prescribing in this population. Over 17,000 individuals with IBD were included in our analyses from a large nationally representative validated research database, free from referral centre bias, allowing us to draw conclusions regarding the UK IBD population. The Misuse of Drugs Regulations 2001 place restrictions on the “schedule 2, 3 and 4 drugs” studied here, meaning over the counter purchase is not possible, and prescription durations are limited. Therefore, we have captured a large proportion of the population‐wide prescribing [65]. Prescriptions were recorded directly at the time of consultation and are therefore not subject to recall bias or selected reporting by patients.

This study did not aim to determine the reasons behind the prescription of pain and sedative medications but instead focuses on their usage, recognising that all long‐term and co‐prescribing carries increased risks, regardless of the underlying indication. As with all observational studies using routinely collected data, there may be missing data and inaccuracies in coding. Regional representation within the UK was uneven and changed over the study period, driven by a transition from the “Vision” computer system, which links to CPRD Gold, to “EMIS”, which integrates with CPRD Aurum. This shift resulted in increased representation of the devolved nations, where Vision remains more prevalent [66, 67]. An IMD result was only available for individuals registered with GP practices in England, and thus, the migration also resulted in missing IMD data within the cohort. The proportion of missing IMD data (84% by 2019) was too large for IMD to be included in the regression analysis.

The lack of a comparison cohort limits our ability to compare prescribing patterns in the IBD cohort with the general population. Furthermore, while patients' risk factors, such as age, diagnosis, and socioeconomic status, evolve over time, we assessed these factors only at the point of diagnosis. This approach was taken to provide clinicians with a framework for identifying and mitigating risks at the earliest stage of care.

### 3.7 Implications Moving Forwards

It is increasingly recognised that an individual's experience of pain is influenced not only by physical factors such as inflammatory burden, bowel damage, surgical complications, and extra‐intestinal manifestations (EIM) [16] but also by external stressors, past experiences, and coping strategies [8, 16]. While pharmacological approaches may play a role in short‐term management of pain and impaired sleep, timely disease control and adequate psychological support are more effective in improving symptoms long‐term [16].

While gabapentinoids may be perceived as safer alternatives to opioids for chronic pain or neuropathic symptoms, their role in IBD remains uncertain. Potential benefits include reduced opioid requirements and treatment of overlapping conditions such as functional abdominal pain or fibromyalgia, which may be more common in IBD. However, gabapentinoids carry their own risks, including sedation, dizziness, and—when co‐prescribed with opioids or other sedatives—an increased risk of respiratory depression and overdose [6, 37]. Their rising use underscores the need for clearer guidance on the management of pain and functional symptoms in IBD, as well as the development of integrated, multidisciplinary approaches that prioritise non‐pharmacological strategies where possible.

Co‐prescribing of pain and sedative medications amplifies the risk of adverse effects [22, 24, 25], necessitating close monitoring and avoidance whenever possible.

The regression analysis identifies individuals at higher risk of harmful prescribing patterns, offering an opportunity to prioritise these patients for targeted interventions. For example, at‐risk individuals could be prioritised for psychological training in coping mechanisms and education on the risks of chronic and co‐prescribed medication use early during treatment to mitigate these risks.

## 4 Conclusion

A substantial proportion of individuals with IBD are prescribed opioid, gabapentinoid, and sedative medications, with co‐prescribing and long‐term use posing considerable risks. This study identified several clinical characteristics that may be used to identify individuals who are at high risk of using these agents.

## Author Contributions

**Samantha Baillie:** conceptualization, methodology, data curation, formal analysis, writing – original draft. **Sonia Saxena:** conceptualization, methodology, formal analysis, supervision, writing – review and editing. **Nishani Jayasooriya:** conceptualization, methodology, data curation, formal analysis, writing – review and editing. **Alex Bottle:** methodology, formal analysis, writing – review and editing. **Irene Petersen:** methodology, writing – review and editing, formal analysis. **Jonathan Blackwell:** methodology, data curation, formal analysis, supervision, writing – review and editing. **Richard Pollok:** conceptualization, methodology, formal analysis, writing – review and editing, supervision.

## Disclosure

The POP‐IBD study group is a collaboration between St George's, University of London, Imperial College London, University College London, and King's College London, conducting population‐based studies in the field of inflammatory bowel disease. All authors contributed to the development of the analysis, interpreting data and preparing the manuscript. R.P. will act as the guarantor for the article.

## Conflicts of Interest

S.B. has had speaker arrangements with Takeda and Dr. Falk, has received a travel grant from Galapagos and Ferring and has provided consultancy to Galapagos. N.J. has had speaker arrangements with Takeda and has received travel grants from Celltrion and Ferring. J.B. has received speaker fees from Dr. Falk, Pfizer, Takeda, Thermo Fisher Scientific, and Ferring. He has provided consultancy to Takeda. R.P. has provided consultancy to Galapagos.

## Supporting information


Data S1

## Data Availability

The data that support the findings of this study are not publicly available and are available from Clinical Research Practice Datalink. CPRD data governance does not allow us to distribute patient data to other parties. Researchers may apply for data access at http://www.CPRD.com/.
