# Supplementary material for: Pain and Sedative Medication Use Among Individuals With Inflammatory Bowel Disease: A Nationwide Population‐Based Cohort Study
Source: Aliment Pharmacol Ther. 2025 Jul 2;62(7):732–42. doi: 10.1111/apt.70247 (PMC12422721; doi:10.1111/apt.70247)
Supplement: Supplementary file 1 — Data S1 [file APT-62-732-s001.docx]

**Supplementary Material**

|  | Year | | | | | | | | | | | | | | | | | | | |
| --- | --- | --- | --- | --- | --- | --- | --- | --- | --- | --- | --- | --- | --- | --- | --- | --- | --- | --- | --- | --- |
|  | 2010 | | 2011 | | 2012 | | 2013 | | 2014 | | 2015 | | 2016 | | 2017 | | 2018 | | 2019 | |
|  | n | % | n | % | n | % | n | % | n | % | n | % | n | % | n | % | n | % | n | % |
| **Total number of individuals** | 15577 | (100.0) | 16435 | (100.0) | 17233 | (100.0) | 17388 | (100.0) | 16626 | (100.0) | 14850 | (100.0) | 13685 | (100.0) | 13222 | (100.0) | 13270 | (100.0) | 13086 | (100.0) |
| **Age** |  |  |  |  |  |  |  |  |  |  |  |  |  |  |  |  |  |  |  |  |
| Median | 52 |  | 52 |  | 52 |  | 52 |  | 52 |  | 52 |  | 52 |  | 52 |  | 53 |  | 53 |  |
| Missing data | 0 | (0.0) | 0 | (0.0) | 0 | (0.0) | 0 | (0.0) | 0 | (0.0) | 0 | (0.0) | 0 | (0.0) | 0 | (0.0) | 0 | (0.0) | 0 | (0.0) |
| **Sex** |  |  |  |  |  |  |  |  |  |  |  |  |  |  |  |  |  |  |  |  |
| Female sex | 7758 | (49.8) | 8212 | (50.0) | 8610 | (50.0) | 8772 | (50.4) | 8403 | (50.5) | 7490 | (50.4) | 6901 | (50.4) | 6679 | (50.5) | 6705 | (50.5) | 6589 | (50.4) |
| Missing data | 0 | (0.0) | 0 | (0.0) | 0 | (0.0) | 0 | (0.0) | 0 | (0.0) | 0 | (0.0) | 0 | (0.0) | 0 | (0.0) | 0 | (0.0) | 0 | (0.0) |
| **IBD Type** |  |  |  |  |  |  |  |  |  |  |  |  |  |  |  |  |  |  |  |  |
| Crohn's disease | 4739 | (30.4) | 5108 | (31.1) | 5363 | (31.1) | 5426 | (31.2) | 5238 | (31.5) | 4735 | (31.9) | 4441 | (32.5) | 4270 | (32.3) | 4321 | (32.6) | 4326 | (33.1) |
| Ulcerative colitis | 10838 | (69.6) | 11327 | (68.9) | 11870 | (68.9) | 11962 | (68.8) | 11388 | (68.5) | 10115 | (68.1) | 9244 | (67.5) | 8952 | (67.7) | 8949 | (67.4) | 8760 | (66.9) |
| **BMI** |  |  |  |  |  |  |  |  |  |  |  |  |  |  |  |  |  |  |  |  |
| (median, kg/m^2^) | 27 |  | 27 |  | 26 |  | 27 |  | 27 |  | 27 |  | 26 |  | 27 |  | 27 |  | 27 |  |
| Missing data | 13542 | (86.9) | 14384 | (87.5) | 15139 | (87.8) | 15409 | (88.6) | 14863 | (89.4) | 13410 | (90.3) | 12545 | (91.7) | 12208 | (92.3) | 12256 | (92.4) | 12145 | (92.8) |
| **Smoking status^†^** |  |  |  |  |  |  |  |  |  |  |  |  |  |  |  |  |  |  |  |  |
| Current | 2925 | (18.8) | 2753 | (16.8) | 2853 | (16.6) | 2896 | (16.7) | 2742 | (16.5) | 2290 | (15.4) | 1919 | (14.0) | 1695 | (12.8) | 1559 | (11.7) | 1427 | (10.9) |
| Previous | 5153 | (33.1) | 5517 | (33.6) | 5767 | (33.5) | 5863 | (33.7) | 5582 | (33.6) | 4845 | (32.6) | 4101 | (30.0) | 3835 | (29.0) | 3862 | (29.1) | 3802 | (29.1) |
| Never | 5651 | (36.3) | 5225 | (31.8) | 5310 | (30.8) | 5282 | (30.4) | 5030 | (30.3) | 4377 | (29.5) | 3692 | (27.0) | 3352 | (25.4) | 3324 | (25.0) | 3226 | (24.7) |
| Missing data | 1848 | (11.9) | 2940 | (17.9) | 3303 | (19.2) | 3347 | (19.2) | 3272 | (19.7) | 3338 | (22.5) | 3973 | (29.0) | 4340 | (32.8) | 4525 | (34.1) | 4631 | (35.4) |
| **IMD^‡^** |  |  |  |  |  |  |  |  |  |  |  |  |  |  |  |  |  |  |  |  |
| 1 | 1512 | (9.7) | 1580 | (9.6) | 1668 | (9.7) | 1511 | (8.7) | 1321 | (7.9) | 936 | (6.3) | 746 | (5.5) | 702 | (5.3) | 631 | (4.8) | 363 | (2.8) |
| 2 | 1513 | (9.7) | 1594 | (9.7) | 1520 | (8.8) | 1569 | (9.0) | 1361 | (8.2) | 986 | (6.6) | 653 | (4.8) | 498 | (3.8) | 474 | (3.6) | 335 | (2.6) |
| 3 | 1507 | (9.7) | 1543 | (9.4) | 1621 | (9.4) | 1554 | (8.9) | 1235 | (7.4) | 852 | (5.7) | 750 | (5.5) | 622 | (4.7) | 508 | (3.8) | 355 | (2.7) |
| 4 | 1633 | (10.5) | 1588 | (9.7) | 1636 | (9.5) | 1478 | (8.5) | 1256 | (7.6) | 919 | (6.2) | 514 | (3.8) | 422 | (3.2) | 422 | (3.2) | 349 | (2.7) |
| 5 | 2008 | (12.9) | 2023 | (12.3) | 1944 | (11.3) | 1804 | (10.4) | 1570 | (9.4) | 1300 | (8.8) | 1053 | (7.7) | 867 | (6.6) | 705 | (5.3) | 674 | (5.2) |
| Missing data | 7404 | (47.5) | 8107 | (49.3) | 8844 | (51.3) | 9472 | (54.5) | 9883 | (59.4) | 9857 | (66.4) | 9969 | (72.8) | 10111 | (76.5) | 10530 | (79.4) | 11010 | (84.1) |
| **Region** |  |  |  |  |  |  |  |  |  |  |  |  |  |  |  |  |  |  |  |  |
| 1 North East | 282 | (1.8) | 272 | (1.7) | 260 | (1.5) | 204 | (1.2) | 97 | (0.6) | 87 | (0.6) | 88 | (0.6) | 0 | (0.0) | 0 | (0.0) | 0 | (0.0) |
| 2 North West | 1850 | (11.9) | 1919 | (11.7) | 1969 | (11.4) | 1924 | (11.1) | 1605 | (9.7) | 1204 | (8.1) | 836 | (6.1) | 796 | (6.0) | 833 | (6.3) | 858 | (6.6) |
| 3 Yorkshire & The Humber | 360 | (2.3) | 261 | (1.6) | 191 | (1.1) | 135 | (0.8) | 149 | (0.9) | 101 | (0.7) | 75 | (0.5) | 18 | (0.1) | 19 | (0.1) | 20 | (0.2) |
| 4 East Midlands | 332 | (2.1) | 251 | (1.5) | 173 | (1.0) | 48 | (0.3) | 1 | (0.0) | 0 | (0.0) | 0 | (0.0) | 0 | (0.0) | 0 | (0.0) | 0 | (0.0) |
| 5 West Midlands | 1188 | (7.6) | 1254 | (7.6) | 1356 | (7.9) | 1313 | (7.6) | 1179 | (7.1) | 778 | (5.2) | 660 | (4.8) | 583 | (4.4) | 542 | (4.1) | 454 | (3.5) |
| 6 East of England | 1012 | (6.5) | 948 | (5.8) | 890 | (5.2) | 785 | (4.5) | 694 | (4.2) | 318 | (2.1) | 292 | (2.1) | 174 | (1.3) | 67 | (0.5) | 44 | (0.3) |
| 7 South West | 1090 | (7.0) | 1162 | (7.1) | 1220 | (7.1) | 1106 | (6.4) | 861 | (5.2) | 631 | (4.2) | 444 | (3.2) | 315 | (2.4) | 243 | (1.8) | 186 | (1.4) |
| 8 South Central | 1493 | (9.6) | 1571 | (9.6) | 1621 | (9.4) | 1674 | (9.6) | 1560 | (9.4) | 1041 | (7.0) | 570 | (4.2) | 396 | (3.0) | 332 | (2.5) | 109 | (0.8) |
| 9 London | 938 | (6.0) | 1027 | (6.2) | 1117 | (6.5) | 1158 | (6.7) | 867 | (5.2) | 607 | (4.1) | 585 | (4.3) | 543 | (4.1) | 441 | (3.3) | 326 | (2.5) |
| 10 South East Coast | 1454 | (9.3) | 1562 | (9.5) | 1600 | (9.3) | 1557 | (9.0) | 1614 | (9.7) | 1609 | (10.8) | 1304 | (9.5) | 1186 | (9.0) | 1135 | (8.6) | 817 | (6.2) |
| 11 Northern Ireland | 847 | (5.4) | 946 | (5.8) | 1023 | (5.9) | 1122 | (6.5) | 1219 | (7.3) | 1299 | (8.7) | 1298 | (9.5) | 1398 | (10.6) | 1485 | (11.2) | 1579 | (12.1) |
| 12 Scotland | 2946 | (18.9) | 3284 | (20.0) | 3624 | (21.0) | 3994 | (23.0) | 4268 | (25.7) | 4579 | (30.8) | 4835 | (35.3) | 4950 | (37.4) | 5163 | (38.9) | 5472 | (41.8) |
| 13 Wales | 1785 | (11.5) | 1978 | (12.0) | 2189 | (12.7) | 2368 | (13.6) | 2512 | (15.1) | 2596 | (17.5) | 2698 | (19.7) | 2863 | (21.7) | 3010 | (22.7) | 3221 | (24.6) |
| Missing data | 0 | (0.0) | 0 | (0.0) | 0 | (0.0) | 0 | (0.0) | 0 | (0.0) | 0 | (0.0) | 0 | (0.0) | 0 | (0.0) | 0 | (0.0) | 0 | (0.0) |
| **Comorbidities^§^** |  |  |  |  |  |  |  |  |  |  |  |  |  |  |  |  |  |  |  |  |
| Anxiety or depression | 3591 | (23.1) | 4002 | (24.4) | 4359 | (25.3) | 4602 | (26.5) | 4653 | (28.0) | 4342 | (29.2) | 4172 | (30.5) | 4174 | (31.6) | 4339 | (32.7) | 4463 | (34.1) |
| Irritable bowel syndrome | 1848 | (11.9) | 1918 | (11.7) | 1983 | (11.5) | 1969 | (11.3) | 1819 | (10.9) | 1486 | (10.0) | 1214 | (8.9) | 1048 | (7.9) | 1036 | (7.8) | 948 | (7.2) |
| Inflammatory arthropathy | 556 | (3.6) | 581 | (3.5) | 601 | (3.5) | 618 | (3.6) | 623 | (3.7) | 571 | (3.8) | 526 | (3.8) | 547 | (4.1) | 558 | (4.2) | 567 | (4.3) |
| Fibromyalgia | 174 | (1.1) | 191 | (1.2) | 210 | (1.2) | 219 | (1.3) | 231 | (1.4) | 226 | (1.5) | 228 | (1.7) | 243 | (1.8) | 272 | (2.0) | 292 | (2.2) |

Table S1

Patient characteristics at the time of inflammatory bowel disease diagnosis, for those diagnosed within the time period 2010-2019

†Smoking status: most recent status recorded in the previous 3 years

‡IMD: Index of multiple deprivation; a measure of the relative deprivation of the area where a GP practice is located with 1 being the least deprived quintile and 5 being the most deprived quintile.

§Comorbidities: individuals with a previous diagnosis of each relevant comorbidity.

Abbreviations: IBD inflammatory bowel disease, BMI body mass index, IMD index of multiple deprivation .

|  | Composite Outcome | | | |
| --- | --- | --- | --- | --- |
|  | Positive | | Negative | |
| Number of individuals | 664 | (100%) | 4237 | (100%) |
| **Age** |  |  |  |  |
| Median, (25^th^-75^th^ percentile) | 54 | (40-67) | 46 | (32-61) |
| Missing data | 0 | (0%) | 0 |  |
| **Sex** |  |  |  |  |
| Female | 390 | (59%) | 2024 | (48%) |
| Male | 274 | (41%) | 2213 | (52%) |
| Missing data | 0 | (0%) | 0 | (0%) |
| **IBD Type** |  |  |  |  |
| Crohn's disease | 240 | (36%) | 1338 | (32%) |
| Ulcerative colitis | 424 | (64%) | 2899 | (68%) |
| **Smoking status^†^** |  |  |  |  |
| Current | 179 | (27%) | 592 | (14%) |
| Previous | 188 | (28%) | 1197 | (28%) |
| Never | 188 | (28%) | 1394 | (33%) |
| Missing data | 109 | (16%) | 1054 | (25%) |
| **Comorbidities^‡^** |  |  |  |  |
| Anxiety or depression | 321 | (48%) | 879 | (21%) |
| Irritable bowel syndrome | 131 | (20%) | 545 | (13%) |
| Inflammatory arthropathy | 33 | (5%) | 96 | (2%) |
| Fibromyalgia | 31 | (5%) | 22 | (1%) |
| Missing data | 0 | (0%) | 0 | (0%) |
| **Steroid use**§ |  |  |  |  |
| Yes | 135 | (20%) | 630 | (15%) |
| No | 529 | (80%) | 3607 | (85%) |
| **Surgery**†† |  |  |  |  |
| Yes | 23 | (3%) | 124 | (3%) |
| No | 641 | (97%) | 4113 | (97%) |
| **Antidepressant use**‡‡ |  |  |  |  |
| Yes | 644 | (97%) | 3697 | (87%) |
| No | 20 | (3%) | 540 | (13%) |

Table S2: Baseline characteristics for individuals with and without the composite outcome (chronic opioid or sedative prescription or co-prescription of sedative/gabapentinoid/strong opioid medication during the 5 year follow up period)

† Smoking status: most recent status recorded in the 2 years prior to diagnosis

‡ Comorbidities: individuals with a previous diagnosis of each relevant comorbidity at the time of inflammatory bowel disease diagnosis

§ Chronic and/or refractory steroid use defined as a steroid prescription lasting more than 56 days and/or refractory use defined as more than one steroid prescription within a one-year period within 30 days of a recorded gastrointestinal symptom.

††IBD-related surgery in the 5 year follow-up period

‡‡ Antidepressant medication use in the year prior to IBD diagnosis

Abbreviations: IBD inflammatory bowel disease

|  |  | Primary Medication | | | | | | | | | | | |
| --- | --- | --- | --- | --- | --- | --- | --- | --- | --- | --- | --- | --- | --- |
|  |  | Any Pain/sedative  Medication | | Strong Opioids | | Weak Opioids | | All Opioids | | Gabapentinoids | | Sedatives^‡^ | |
| Year | Total† | n= | (%) | n= | (%) | n= | (%) | n= | (%) | n= | (%) | n= | (%) |
| 2019 | 13086 | 693 | (5.3) | 489 | (3.7) | 386 | (2.9) | 621 | (4.7) | 411 | (3.1) | 427 | (3.3) |
| 2018 | 13270 | 732 | (5.5) | 520 | (3.9) | 416 | (3.2) | 662 | (5.0) | 413 | (3.1) | 449 | (3.4) |
| 2017 | 13222 | 740 | (5.6) | 536 | (4.1) | 412 | (3.1) | 668 | (5.1) | 419 | (3.2) | 446 | (3.4) |
| 2016 | 13685 | 943 | (6.9) | 586 | (4.3) | 466 | (3.5) | 864 | (6.3) | 407 | (3.0) | 436 | (3.2) |
| 2015 | 14850 | 849 | (5.7) | 629 | (4.2) | 529 | (3.9) | 772 | (5.2) | 474 | (3.2) | 509 | (3.4) |
| 2014 | 16626 | 898 | (5.4) | 663 | (4.0) | 595 | (4.0) | 823 | (5.0) | 465 | (2.8) | 547 | (3.3) |
| 2013 | 17388 | 854 | (4.9) | 639 | (3.7) | 566 | (3.4) | 796 | (4.6) | 402 | (2.3) | 567 | (3.3) |
| 2012 | 17233 | 783 | (4.5) | 611 | (3.5) | 512 | (2.9) | 738 | (4.3) | 343 | (2.0) | 513 | (3.0) |
| 2011 | 16435 | 699 | (4.3) | 532 | (3.2) | 478 | (2.8) | 661 | (4.0) | 278 | (1.7) | 485 | (3.0) |
| 2010 | 15577 | 651 | (4.2) | 490 | (3.1) | 436 | (2.7) | 610 | (3.9) | 216 | (1.4) | 475 | (3.0) |

Table S3: Annual prevalence of co-prescribed medication 2010-2019

Total number and % of individuals with a co-prescribed medication (i.e. co-prescription of opioid plus gabapentinoid or sedative, gabapentinoid plus opioid or sedative, and sedative plus opioid or gabapentinoid)

| † Total number of patients in the follow up cohort in the given year | | | | | | | | | |
| --- | --- | --- | --- | --- | --- | --- | --- | --- | --- |
| ‡Benzodiazepines and Z-drugs combined |  |  |  |  |  |  |  |  |  |

|  | Annual Prevalence | | | |
| --- | --- | --- | --- | --- |
|  | All opioids^†^ | | All sedatives^‡^ | |
| Year | Total | Chronic ^§^ | Total | Chronic^¶^ |
| 2019 | 14.0 | 4.8 | 8.4 | 4.4 |
| 2018 | 14.1 | 5.2 | 8.6 | 4.4 |
| 2017 | 14.6 | 5.5 | 8.3 | 4.0 |
| 2016 | 15.4 | 5.7 | 8.5 | 4.2 |
| 2015 | 15.8 | 6.0 | 9.1 | 4.7 |
| 2014 | 15.9 | 6.2 | 9.6 | 4.6 |
| 2013 | 15.4 | 6.2 | 9.4 | 4.5 |
| 2012 | 14.2 | 6.0 | 9.0 | 4.3 |
| 2011 | 13.1 | 5.8 | 8.7 | 4.2 |
| 2010 | 13.6 | 5.9 | 8.4 | 4.2 |

Table S4 The number of patients per 100 receiving a prescription for opioids or sedatives per year 2010-2019

† strong and weak opioids

‡ benzodiazepines and Z-drugs

§ more than 90 days continuous prescription

¶ more than 28 days continuous prescription


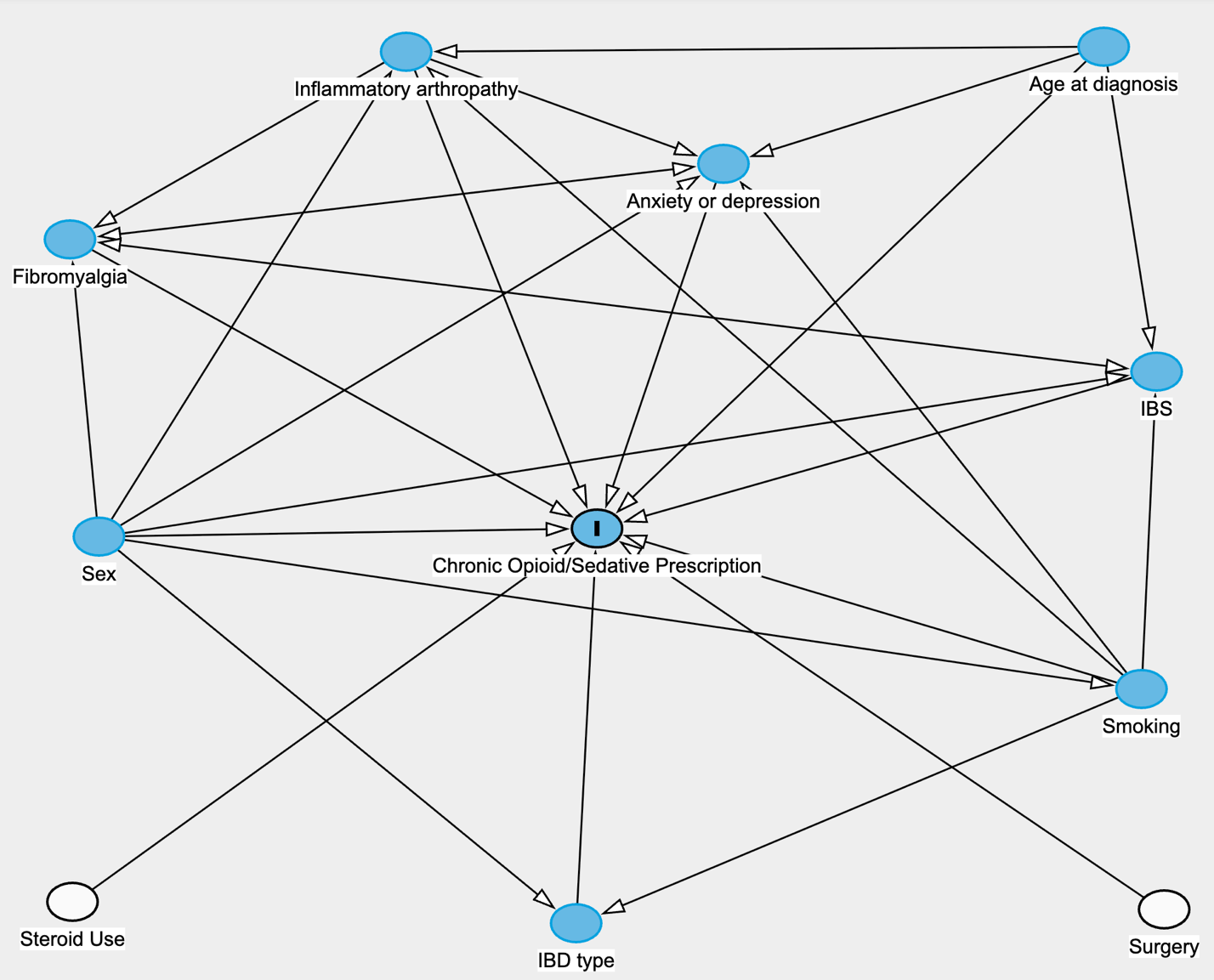


Figure S1

Direct Acyclic Graph of interactions between predictors, covariates and outcome (chronic/co-prescribing)

**Crohn’s Disease Code List**

| **Medcode** | **Description** |
| --- | --- |
| 593 | Crohn's disease |
| 6538 | Crohn's colitis |
| 9359 | Crohn's disease of the small bowel NOS |
| 11286 | Regional enteritis - Crohn's disease |
| 15773 | Regional ileocolitis |
| 20688 | Crohn's disease of the large bowel NOS |
| 28476 | Crohn's disease of the terminal ileum |
| 39278 | Crohn's disease of the ileum NOS |
| 44426 | Regional enteritis of the large bowel |
| 51576 | Regional enteritis of the small bowel |
| 51578 | Granulomatous enteritis |
| 59994 | Crohn's disease NOS |
| 62628 | Regional enteritis of the colon |
| 63036 | Regional enteritis of the jejunum |
| 64773 | Regional enteritis of the rectum |
| 66238 | Crohn's disease of the ileum unspecified |
| 71945 | Regional enteritis of the duodenum |

**Ulcerative Colitis Code List**

| **Medcode** | **Description** |
| --- | --- |
| 704 | Ulcerative colitis |
| 1784 | Ulcerative colitis and/or proctitis |
| 1795 | Proctitis NOS |
| 5749 | H/O: ulcerative colitis |
| 6650 | Ulcerative proctocolitis |
| 8347 | Ulcerative proctitis |
| 15207 | Idiopathic proctocolitis NOS |
| 24550 | Other idiopathic proctocolitis |
| 24858 | Ulcerative rectosigmoiditis |
| 30433 | Ulcerative (chronic) enterocolitis |
| 33456 | Ulcerative proctocolitis NOS |
| 42822 | Ulcerative (chronic) ileocolitis |
| 43090 | Other idiopathic proctocolitis NOS |
| 48732 | Ulcerative ileocolitis |
| 104259 | Ulcerative pancolitis |

**Weak Opioids Code List**

| **Prodcode** | | **Drug Substance Name** |  |
| --- | --- | --- | --- |
| 4 | Dextropropoxyphene Hydrochloride/Paracetamol | | |
| 53 | Dihydrocodeine tartrate | | |
| 86 | Tramadol Hydrochloride | | |
| 124 | Dextropropoxyphene Hydrochloride/Paracetamol | | |
| 152 | Codeine phosphate | | |
| 158 | Codeine phosphate | | |
| 187 | Tramadol Hydrochloride | | |
| 191 | Dihydrocodeine tartrate | | |
| 213 | Codeine phosphate | | |
| 382 | Codeine phosphate | | |
| 462 | Codeine phosphate | | |
| 539 | Codeine phosphate | | |
| 635 | Codeine phosphate | | |
| 687 | Paracetamol/Tramadol Hydrochloride | | |
| 701 | Tramadol Hydrochloride | | |
| 913 | Codeine phosphate/Kaolin light | | |
| 1617 | Codeine phosphate | | |
| 2041 | Dihydrocodeine tartrate | | |
| 2555 | Dihydrocodeine Tartrate/Paracetamol | | |
| 2764 | Codeine phosphate | | |
| 3378 | Tramadol Hydrochloride | | |
| 3644 | Tramadol Hydrochloride | | |
| 3653 | Dihydrocodeine tartrate | | |
| 3714 | Dextropropoxyphene Napsylate/Paracetamol | | |
| 4114 | Tramadol Hydrochloride | | |
| 4115 | Tramadol Hydrochloride | | |
| 4369 | Codeine phosphate | | |
| 4805 | Codeine Phosphate | | |
| 4823 | Dihydrocodeine tartrate | | |
| 4834 | Tramadol Hydrochloride | | |
| 4950 | Dihydrocodeine Tartrate/Paracetamol | | |
| 4999 | Tramadol 24 Modified-Release Tablet | | |
| 5028 | Tramadol 24 Modified-Release Tablet | | |
| 5169 | Tramadol Hydrochloride | | |
| 5257 | Tramadol 12 Modified-Release Tablet | | |
| 5572 | Codeine phosphate | | |
| 6153 | Tramadol Hydrochloride | | |
| 6215 | Tramadol Hydrochloride | | |
| 6234 | Dihydrocodeine tartrate | | |
| 6558 | Paracetamol/Tramadol Hydrochloride | | |
| 7950 | Doloxene 65 Mg Pul | | |
| 7989 | Dihydrocodeine Tartrate/Aspirin 300 Mg Tab | | |
| 8233 | Kaolin Light 1.5G/5Ml / Codeine 5Mg/5Ml Oral Suspension Sugar Free | | |
| 8416 | Tramadol 12 Modified-Release Tablet | | |
| 8456 | Dihydrocodeine tartrate | | |
| 9044 | Aspirin/Codeine phosphate | | |
| 9209 | Dihydrocodeine tartrate | | |
| 9275 | Dihydrocodeine tartrate | | |
| 9313 | Dihydrocodeine tartrate | | |
| 9389 | Tramadol Hydrochloride | | |
| 9396 | Tramadol Hydrochloride | | |
| 9739 | Tramadol Hydrochloride | | |
| 10023 | Dihydrocodeine Tartrate/Paracetamol | | |
| 10122 | Dihydrocodeine Tartrate/Paracetamol | | |
| 11101 | Tramadol Hydrochloride | | |
| 11275 | Tramadol | | |
| 11471 | Tramadol | | |
| 11549 | Tramadol Hydrochloride | | |
| 11559 | Tramadol Hydrochloride | | |
| 11734 | Tramadol Hydrochloride | | |
| 11746 | Tramadol Hydrochloride | | |
| 11748 | Tramadol Hydrochloride | | |
| 12076 | Dextropropoxyphene 60Mg Capsules | | |
| 12992 | Aspirin/Codeine phosphate | | |
| 13813 | Tramadol Hydrochloride | | |
| 14490 | Tramadol Hydrochloride | | |
| 15360 | Codeine phosphate | | |
| 16096 | Codeine phosphate | | |
| 16271 | Tramadol Hydrochloride | | |
| 16395 | Tramadol Hydrochloride | | |
| 17917 | Dihydrocodeine Tartrate/Paracetamol | | |
| 19069 | Dextropropoxyphene | | |
| 19993 | Tramadol Hydrochloride | | |
| 20310 | Tramadol Hydrochloride | | |
| 21113 | Dihydrocodeine Tartrate/Paracetamol | | |
| 21256 | Tramadol Hydrochloride | | |
| 21397 | Tramadol Hydrochloride | | |
| 21777 | Tramadol Hydrochloride | | |
| 21797 | Tramadol Hydrochloride | | |
| 21947 | Tramadol Hydrochloride | | |
| 23625 | Tramadol Hydrochloride | | |
| 23977 | Codeine phosphate | | |
| 23981 | Tramadol Hydrochloride | | |
| 24124 | Codeine phosphate | | |
| 24125 | Codeine phosphate | | |
| 24383 | Tramadol Hydrochloride | | |
| 24733 | Dextropropoxyphene Hydrochloride | | |
| 25485 | Codeine Phosphate | | |
| 25959 | Aspirin/Caffeine/Dextropropoxyphene | | |
| 25979 | Dextropropoxyphene | | |
| 26336 | Tramadol Hydrochloride | | |
| 26653 | Dihydrocodeine Paracetamol | | |
| 26986 | Tramadol Hydrochloride | | |
| 27591 | Tramadol Hydrochloride | | |
| 28728 | Tramadol Hydrochloride | | |
| 29324 | Tramadol Hydrochloride | | |
| 29860 | Tramadol Hydrochloride | | |
| 30295 | Dihydrocodeine Tartrate/Paracetamol | | |
| 30954 | Dextropropoxyphene Hydrochloride/Paracetamol | | |
| 31105 | Tramadol Hydrochloride | | |
| 31107 | Tramadol Hydrochloride | | |
| 31452 | Codeine phosphate | | |
| 31700 | Codeine phosphate | | |
| 31734 | Tramadol Hydrochloride | | |
| 31943 | Codeine phosphate | | |
| 32165 | Tramadol hydrochloride | | |
| 32436 | Codeine phosphate | | |
| 32450 | Tramadol Hydrochloride | | |
| 33528 | Codeine phosphate | | |
| 33647 | Dextropropoxyphene hydrochloride/Paracetamol | | |
| 33654 | Dihydrocodeine tartrate | | |
| 33743 | Dihydrocodeine With Paracetamol Effervescent Tablets | | |
| 34008 | Dihydrocodeine tartrate | | |
| 34022 | Dextropropoxyphene Hydrochloride/Paracetamol | | |
| 34065 | Tramadol Hydrochloride | | |
| 34090 | Codeine phosphate | | |
| 34099 | Codeine phosphate | | |
| 34152 | Codeine phosphate | | |
| 34168 | Codeine phosphate | | |
| 34172 | Codeine phosphate | | |
| 34176 | Codeine phosphate | | |
| 34260 | Tramadol hydrochloride | | |
| 34281 | Tramadol Hydrochloride | | |
| 34319 | Dextropropoxyphene hydrochloride/Paracetamol | | |
| 34348 | Codeine phosphate | | |
| 34349 | Dextropropoxyphene hydrochloride/Paracetamol | | |
| 34373 | Codeine phosphate | | |
| 34383 | Codeine phosphate | | |
| 34422 | Tramadol hydrochloride | | |
| 34437 | Codeine phosphate | | |
| 34440 | Dihydrocodeine tartrate | | |
| 34444 | Codeine phosphate | | |
| 34468 | Dextropropoxyphene hydrochloride/Paracetamol | | |
| 34521 | Tramadol hydrochloride | | |
| 34552 | Codeine phosphate | | |
| 34570 | Tramadol hydrochloride | | |
| 34579 | Dihydrocodeine tartrate | | |
| 34597 | Dextropropoxyphene hydrochloride/Paracetamol | | |
| 34639 | Tramadol hydrochloride | | |
| 34662 | Dihydrocodeine tartrate | | |
| 34730 | Dihydrocodeine tartrate | | |
| 34789 | Codeine phosphate | | |
| 34808 | Tramadol hydrochloride | | |
| 35347 | Tramadol Hydrochloride | | |
| 35438 | Tramadol hydrochloride | | |
| 35651 | Tramadol hydrochloride | | |
| 35656 | Tramadol hydrochloride | | |
| 35792 | Codeine phosphate | | |
| 35806 | Tramadol hydrochloride | | |
| 36035 | Tramadol hydrochloride | | |
| 36697 | Tramadol hydrochloride | | |
| 36732 | Tramadol hydrochloride | | |
| 36873 | Tramadol hydrochloride | | |
| 36949 | Tramadol hydrochloride | | |
| 37020 | Tramadol hydrochloride | | |
| 37021 | Tramadol hydrochloride | | |
| 37831 | Tramadol hydrochloride | | |
| 37867 | Tramadol Hydrochloride | | |
| 38196 | Tramadol hydrochloride | | |
| 38521 | Dihydrocodeine tartrate | | |
| 38528 | Tramadol hydrochloride | | |
| 38874 | Tramadol hydrochloride | | |
| 38956 | Tramadol hydrochloride | | |
| 38970 | Dihydrocodeine tartrate | | |
| 38987 | Codeine phosphate | | |
| 39505 | Tramadol hydrochloride | | |
| 39558 | Dihydrocodeine tartrate | | |
| 39709 | Tramadol hydrochloride | | |
| 39750 | Tramadol hydrochloride | | |
| 39798 | Tramadol hydrochloride | | |
| 39811 | Tramadol hydrochloride | | |
| 40058 | Tramadol hydrochloride | | |
| 40060 | Tramadol hydrochloride | | |
| 40061 | Tramadol hydrochloride | | |
| 40159 | Dihydrocodeine tartrate | | |
| 40166 | Tramadol hydrochloride | | |
| 40249 | Tramadol hydrochloride | | |
| 40254 | Tramadol hydrochloride | | |
| 40718 | Tramadol hydrochloride | | |
| 40805 | Tramadol hydrochloride | | |
| 40883 | Tramadol hydrochloride | | |
| 40926 | Tramadol hydrochloride | | |
| 41214 | Codeine phosphate | | |
| 41407 | Dextropropoxyphene hydrochloride/Paracetamol | | |
| 41416 | Codeine phosphate | | |
| 41523 | Codeine phosphate | | |
| 41535 | Codeine phosphate | | |
| 41599 | Codeine phosphate | | |
| 41976 | Tramadol hydrochloride | | |
| 42280 | Paracetamol/Tramadol hydrochloride | | |
| 42332 | Paracetamol/Tramadol hydrochloride | | |
| 42792 | Codeine phosphate | | |
| 42798 | Tramadol hydrochloride | | |
| 43198 | Tramadol hydrochloride | | |
| 43504 | Codeine phosphate | | |
| 43513 | Tramadol hydrochloride | | |
| 43550 | Codeine phosphate | | |
| 43891 | Paracetamol/Dextropropoxyphene hydrochloride | | |
| 44311 | Dextropropoxyphene Napsylate/Aspirin | | |
| 44371 | Tramadol hydrochloride | | |
| 46279 | Tramadol hydrochloride | | |
| 46587 | Tramadol hydrochloride | | |
| 46643 | Tramadol hydrochloride | | |
| 47003 | Codeine phosphate | | |
| 47854 | Tramadol Hydrochloride | | |
| 47919 | Codeine phosphate | | |
| 47952 | Codeine phosphate | | |
| 48004 | Codeine phosphate | | |
| 48066 | Codeine Phosphate | | |
| 48090 | Tramadol hydrochloride | | |
| 48133 | Dihydrocodeine tartrate | | |
| 48136 | Codeine phosphate | | |
| 48153 | Codeine phosphate | | |
| 48964 | Codeine phosphate | | |
| 49323 | Tramadol hydrochloride | | |
| 49324 | Tramadol hydrochloride | | |
| 50421 | Codeine phosphate | | |
| 50468 | Codeine phosphate | | |
| 50532 | Dihydrocodeine tartrate | | |
| 50659 | Codeine phosphate | | |
| 50862 | Tramadol hydrochloride | | |
| 50947 | Tramadol hydrochloride | | |
| 51327 | Codeine phosphate | | |
| 51644 | Codeine phosphate | | |
| 51937 | Codeine phosphate | | |
| 52495 | Tramadol hydrochloride | | |
| 52605 | Tramadol hydrochloride | | |
| 52888 | Codeine phosphate | | |
| 52929 | Codeine phosphate | | |
| 52977 | Tramadol hydrochloride | | |
| 53208 | Dextropropoxyphene hydrochloride/Paracetamol | | |
| 53600 | Codeine phosphate | | |
| 53999 | Codeine phosphate | | |
| 54023 | Tramadol hydrochloride | | |
| 54354 | Dihydrocodeine tartrate | | |
| 55245 | Dextropropoxyphene hydrochloride/Paracetamol | | |
| 55309 | Codeine phosphate | | |
| 55425 | Dihydrocodeine Tartrate | | |
| 56178 | Codeine phosphate | | |
| 56205 | Caffeine/Codeine phosphate/Paracetamol | | |
| 56491 | Tramadol hydrochloride | | |
| 56559 | Codeine phosphate | | |
| 56817 | Codeine phosphate | | |
| 57381 | Codeine phosphate | | |
| 57487 | Codeine phosphate | | |
| 57752 | Codeine phosphate | | |
| 58129 | Tramadol hydrochloride | | |
| 58131 | Codeine phosphate | | |
| 58316 | Tramadol hydrochloride | | |
| 58909 | Codeine phosphate | | |
| 59978 | Dihydrocodeine tartrate | | |
| 59989 | Dihydrocodeine tartrate | | |
| 60121 | Tramadol hydrochloride | | |
| 60489 | Codeine phosphate | | |
| 60640 | Codeine phosphate | | |
| 60751 | Tramadol hydrochloride | | |
| 60958 | Codeine phosphate | | |
| 61049 | Codeine phosphate | | |
| 61091 | Codeine phosphate | | |
| 61272 | Tramadol hydrochloride | | |
| 61610 | Tramadol hydrochloride | | |
| 61775 | Tramadol hydrochloride | | |
| 62228 | Codeine phosphate | | |
| 62778 | Paracetamol/Tramadol hydrochloride | | |
| 63047 | Tramadol hydrochloride | | |
| 63547 | Codeine phosphate | | |
| 63600 | Codeine phosphate | | |
| 63898 | Tramadol hydrochloride | | |
| 64079 | Dihydrocodeine tartrate | | |
| 64108 | Codeine phosphate | | |
| 64368 | Dihydrocodeine tartrate/Paracetamol | | |
| 64459 | Paracetamol/Tramadol hydrochloride | | |
| 64496 | Tramadol hydrochloride | | |
| 64731 | Tramadol hydrochloride | | |
| 64751 | Codeine phosphate | | |
| 64752 | Codeine phosphate | | |
| 64871 | Tramadol hydrochloride | | |
| 65118 | Codeine phosphate | | |
| 65245 | Codeine phosphate | | |
| 65266 | Tramadol hydrochloride | | |
| 65269 | Codeine phosphate | | |
| 65689 | Dihydrocodeine tartrate | | |
| 65954 | Tramadol hydrochloride | | |
| 66115 | Codeine phosphate | | |
| 66121 | Dihydrocodeine tartrate | | |
| 66299 | Tramadol hydrochloride | | |
| 66729 | Tramadol hydrochloride | | |
| 66893 | Codeine phosphate | | |
| 67161 | Tramadol hydrochloride | | |
| 67197 | Tramadol hydrochloride | | |
| 67310 | Tramadol hydrochloride | | |
| 67323 | Tramadol hydrochloride | | |
| 67744 | Tramadol hydrochloride | | |
| 67751 | Codeine phosphate | | |
| 67755 | Dextropropoxyphene hydrochloride/Paracetamol | | |
| 68210 | Tramadol hydrochloride | | |
| 68427 | Tramadol hydrochloride | | |
| 68538 | Codeine phosphate | | |
| 68833 | Tramadol hydrochloride | | |
| 68861 | Codeine phosphate | | |
| 69043 | Dextropropoxyphene hydrochloride/Paracetamol | | |
| 69066 | Codeine phosphate | | |
| 69534 | Dextropropoxyphene hydrochloride/Paracetamol | | |
| 69576 | Codeine phosphate | | |
| 69894 | Tramadol hydrochloride | | |
| 71355 | Tramadol hydrochloride | | |
| 71358 | Tramadol hydrochloride | | |
| 71492 | Codeine phosphate | | |
| 71987 | Codeine phosphate | | |
| 72209 | Dihydrocodeine tartrate | | |
| 72210 | Dihydrocodeine tartrate | | |
| 72265 | Dihydrocodeine tartrate | | |
| 72438 | Codeine phosphate | | |
| 72646 | Tramadol hydrochloride | | |
| 72796 | Dextropropoxyphene hydrochloride/Paracetamol | | |
| 72952 | Codeine phosphate | | |
| 73069 | Tramadol hydrochloride | | |
| 73076 | Tramadol hydrochloride | | |
| 73145 | Dihydrocodeine tartrate | | |
| 73147 | Dihydrocodeine tartrate | | |
| 73306 | Tramadol hydrochloride | | |
| 73336 | Tramadol hydrochloride | | |
| 73596 | Tramadol hydrochloride | | |
| 73631 | Codeine phosphate | | |
| 73890 | Tramadol hydrochloride | | |
| 73923 | Tramadol hydrochloride | | |
| 73924 | Tramadol hydrochloride | | |
| 74002 | Tramadol hydrochloride | | |
| 74247 | Codeine phosphate | | |
| 74498 | Tramadol hydrochloride | | |
| 74830 | Tramadol hydrochloride | | |
| 74839 | Tramadol hydrochloride | | |
| 74887 | Tramadol hydrochloride | | |
| 75286 | Tramadol hydrochloride | | |
| 75304 | Tramadol hydrochloride | | |
| 75502 | Tramadol hydrochloride | | |
| 75661 | Tramadol hydrochloride | | |
| 75864 | Tramadol hydrochloride | | |
| 75930 | Tramadol hydrochloride | | |
| 75931 | Tramadol hydrochloride | | |
| 75958 | Tramadol hydrochloride | | |
| 76224 | Tramadol hydrochloride | | |
| 77034 | Tramadol hydrochloride | | |
| 77356 | Tramadol hydrochloride | | |
| 77485 | Tramadol hydrochloride | | |
| 77505 | Dihydrocodeine tartrate/Paracetamol | | |
| 77642 | Tramadol hydrochloride | | |
| 78049 | Codeine phosphate | | |
| 78059 | Tramadol hydrochloride | | |
| 78150 | Tramadol hydrochloride | | |
| 78761 | Tramadol hydrochloride | | |
| 79001 | Tramadol hydrochloride | | |
| 79594 | Codeine phosphate | | |
| 80001 | Tramadol hydrochloride | | |
| 80005 | Tramadol hydrochloride | | |
| 80049 | Tramadol hydrochloride | | |
| 80464 | Tramadol hydrochloride | | |
| 80809 | Tramadol hydrochloride | | |
| 81145 | Dihydrocodeine | | |
| 81304 | Codeine phosphate | | |
| 81589 | Dihydrocodeine tartrate | | |
| 81683 | Tramadol hydrochloride/Paracetamol | | |
| 82165 | Codeine phosphate | | |
| 82307 | Paracetamol/Tramadol hydrochloride | | |
| 82535 | Tramadol hydrochloride | | |
| 82648 | Tramadol hydrochloride | | |
| 86140 | Dihydrocodeine | | |
| 51937 | Codeine phosphate | | |

**Strong Opioids Code List**

| **Prodcode** | | **Drug Substance Name** |  |
| --- | --- | --- | --- |
| 123 | Pentazocine Lactate | | |
| 148 | Morphine | | |
| 166 | Papaveretum | | |
| 231 | Omnopon | | |
| 234 | Pethidine Hydrochloride | | |
| 249 | Pethidine Hydrochloride | | |
| 320 | Buprenorphine Hydrochloride | | |
| 328 | Pentazocine hydrochloride | | |
| 354 | Morphine Sulphate | | |
| 396 | Buprenorphine hydrochloride | | |
| 423 | Pethidine Hydrochloride | | |
| 458 | Morphine Sulphate | | |
| 495 | Morphine sulphate | | |
| 607 | Morphine sulphate | | |
| 617 | Fentanyl Citrate | | |
| 620 | Fentanyl | | |
| 655 | Morphine sulphate | | |
| 659 | Morphine Sulphate | | |
| 685 | Aspirin/Papaveretum | | |
| 715 | Morphine Sulphate | | |
| 748 | Fentanyl | | |
| 757 | Fentanyl | | |
| 826 | Pethidine hydrochloride | | |
| 1503 | Morphine sulphate | | |
| 2367 | Pentazocine hydrochloride | | |
| 2450 | Pethidine hydrochloride | | |
| 2957 | Morphine sulphate | | |
| 2966 | Pethidine Hydrochloride | | |
| 2997 | Morphine sulphate | | |
| 3064 | Buprenorphine hydrochloride | | |
| 3165 | Diamorphine Hydrochloride | | |
| 3239 | Meptazinol hydrochloride | | |
| 3522 | Buprenorphine hydrochloride | | |
| 3919 | Morphine sulphate | | |
| 3990 | Dextromoramide tartrate | | |
| 4236 | Dextromoramide tartrate | | |
| 4266 | Morphine sulphate | | |
| 4280 | Morphine sulphate | | |
| 4476 | Morphine sulphate | | |
| 4477 | Morphine sulphate | | |
| 4691 | Fentanyl | | |
| 4693 | Morphine sulphate | | |
| 5048 | Fentanyl | | |
| 5079 | Diamorphine Hydrochloride | | |
| 5137 | Hydromorphone hydrochloride | | |
| 5138 | Hydromorphone hydrochloride | | |
| 5555 | Morphine sulphate | | |
| 5563 | Morphine Sulphate | | |
| 5585 | Oxycodone hydrochloride | | |
| 5599 | Oxycodone hydrochloride | | |
| 5651 | Fentanyl citrate | | |
| 5652 | Morphine Sulphate | | |
| 5657 | Fentanyl | | |
| 5664 | Morphine hydrochloride | | |
| 5668 | Diamorphine Hydrochloride | | |
| 5670 | Diamorphine Hydrochloride | | |
| 5681 | Morphine sulphate | | |
| 5696 | Fentanyl citrate | | |
| 5697 | Fentanyl citrate | | |
| 5714 | Morphine sulphate | | |
| 5833 | Morphine tartrate/Cyclizine tartrate | | |
| 5840 | Morphine sulphate | | |
| 5843 | Oxycodone hydrochloride | | |
| 5936 | Buprenorphine | | |
| 5991 | Morphine sulphate | | |
| 6002 | Morphine sulphate | | |
| 6040 | Buprenorphine | | |
| 6056 | Buprenorphine hydrochloride | | |
| 6181 | Buprenorphine | | |
| 6210 | Buprenorphine hydrochloride | | |
| 6226 | Aspirin/Papaveretum | | |
| 6232 | Morphine sulphate | | |
| 6269 | Morphine sulphate | | |
| 6298 | Fentanyl | | |
| 6366 | Morphine sulphate | | |
| 6414 | Oxycodone Hydrochloride | | |
| 6458 | Diamorphine Hydrochloride | | |
| 6459 | Diamorphine Hydrochloride | | |
| 6547 | Buprenorphine hydrochloride | | |
| 6557 | Oxycodone hydrochloride | | |
| 6608 | Oxycodone hydrochloride | | |
| 6609 | Oxycodone hydrochloride | | |
| 6708 | Oxycodone hydrochloride | | |
| 6736 | Morphine sulphate | | |
| 6769 | Oxycodone hydrochloride | | |
| 6790 | Oxycodone hydrochloride | | |
| 6879 | Buprenorphine | | |
| 6892 | Morphine Sulphate | | |
| 6917 | Buprenorphine | | |
| 6948 | Oxycodone hydrochloride | | |
| 7082 | Fentanyl | | |
| 7107 | Fentanyl | | |
| 7114 | Diamorphine Hydrochloride | | |
| 7126 | Fentanyl | | |
| 7167 | Oxycodone hydrochloride | | |
| 7197 | Morphine Sulphate | | |
| 7236 | Buprenorphine | | |
| 7238 | Buprenorphine | | |
| 7275 | Oxycodone hydrochloride | | |
| 7334 | Buprenorphine | | |
| 7372 | Oxycodone hydrochloride | | |
| 7389 | Oxycodone hydrochloride | | |
| 7397 | Fentanyl | | |
| 7406 | Oxycodone hydrochloride | | |
| 7457 | Buprenorphine Hydrochloride | | |
| 7517 | Morphine sulphate | | |
| 7555 | Buprenorphine | | |
| 7729 | Morphine Hydrochloride | | |
| 7800 | Dextromoramide Tartrate | | |
| 7801 | Morphine Sulphate | | |
| 7849 | Diamorphine Hydrochloride | | |
| 7872 | Morphine S/R | | |
| 7875 | Morphine sulphate | | |
| 7998 | Dextromoramide tartrate | | |
| 7999 | Diamorphine Hydrochloride | | |
| 8017 | Buprenorphine hydrochloride | | |
| 8039 | Morphine sulphate | | |
| 8040 | Diamorphine Hydrochloride | | |
| 8075 | Morphine Sulphate | | |
| 8220 | Morphine Anhydrous | | |
| 8375 | Pentazocine hydrochloride | | |
| 8420 | Morphine Sulphate | | |
| 8447 | Meptazinol hydrochloride | | |
| 8460 | Diamorphine | | |
| 8735 | Diamorphine hydrochloride | | |
| 8740 | Morphine hydrochloride | | |
| 8766 | Morphine hydrochloride | | |
| 8822 | Morphine sulphate | | |
| 8823 | Diamorphine Hydrochloride | | |
| 8866 | Diamorphine hydrochloride | | |
| 8867 | Morphine Sulphate Cr | | |
| 8876 | Morphine sulphate | | |
| 8959 | Morphine Sulphate | | |
| 9001 | Cyclizine hydrochloride/Dipipanone hydrochloride | | |
| 9012 | Morphine tartrate/Cyclizine tartrate | | |
| 9053 | Diamorphine Hydrochloride | | |
| 9126 | Diamorphine | | |
| 9137 | Morphine sulphate | | |
| 9183 | Morphine sulphate | | |
| 9325 | Hydromorphone hydrochloride | | |
| 9330 | Hydromorphone hydrochloride | | |
| 9331 | Hydromorphone hydrochloride | | |
| 9332 | Hydromorphone hydrochloride | | |
| 9337 | Morphine sulphate | | |
| 9342 | Morphine sulphate | | |
| 9371 | Morphine sulphate | | |
| 9381 | Morphine sulphate | | |
| 9484 | Morphine Sulphate | | |
| 9557 | Morphine sulphate | | |
| 9602 | Morphine sulphate | | |
| 9615 | Hydromorphone hydrochloride | | |
| 9672 | Morphine sulphate | | |
| 9874 | Oxycodone hydrochloride | | |
| 9927 | Oxycodone hydrochloride | | |
| 9928 | Oxycodone Hydrochloride | | |
| 9945 | Diamorphine hydrochloride | | |
| 9960 | Morphine Sulphate | | |
| 9973 | Oxycodone hydrochloride | | |
| 10021 | Oxycodone hydrochloride | | |
| 10077 | Buprenorphine hydrochloride | | |
| 10205 | Buprenorphine | | |
| 10239 | Morphine sulphate | | |
| 10280 | Omnopon | | |
| 10473 | Diamorphine | | |
| 10525 | Morphine sulphate | | |
| 10578 | Dextromoramide tartrate | | |
| 10583 | Pentazocine Lactate | | |
| 10631 | Morphine Anhydrous | | |
| 10730 | Morphine sulphate | | |
| 10769 | Pentazocine hydrochloride | | |
| 10829 | Morphine sulphate | | |
| 10866 | Oxycodone hydrochloride | | |
| 10907 | Morphine sulphate | | |
| 10922 | Fentanyl | | |
| 10925 | Meptazinol hydrochloride | | |
| 11129 | Papaveretum | | |
| 11342 | Morphine sulphate | | |
| 11405 | Oxycodone hydrochloride | | |
| 11584 | Buprenorphine | | |
| 11698 | Morphine Sulphate | | |
| 11755 | Morphine sulphate | | |
| 11801 | Meptazinol hydrochloride | | |
| 11838 | Morphine sulphate | | |
| 11843 | Fentanyl citrate | | |
| 11971 | Morphine hydrochloride/Cocaine hydrochloride/Syrup/Ethanol 90% | | |
| 11982 | Fentanyl | | |
| 12011 | Morphine sulphate | | |
| 12020 | Cyclizine hydrochloride/Dipipanone hydrochloride | | |
| 12219 | Morphine sulphate | | |
| 12508 | Morphine sulphate | | |
| 12567 | Pentazocine | | |
| 12583 | Morphine sulphate | | |
| 12591 | Morphine sulphate | | |
| 12602 | Morphine sulphate | | |
| 12604 | Morphine sulphate | | |
| 12608 | Morphine sulphate | | |
| 12889 | Morphine sulphate | | |
| 12900 | Morphine sulphate | | |
| 13031 | Buprenorphine hydrochloride | | |
| 13076 | Fentanyl citrate | | |
| 13114 | Morphine sulphate | | |
| 13117 | Morphine sulphate | | |
| 13172 | Morphine sulphate | | |
| 13225 | Morphine sulphate | | |
| 13280 | Morphine sulphate | | |
| 13300 | Buprenorphine | | |
| 13364 | Diamorphine | | |
| 13420 | Diamorphine hydrochloride | | |
| 13423 | Morphine sulphate | | |
| 13588 | Morphine sulphate | | |
| 13711 | Morphine Sulphate | | |
| 13995 | Morphine Sulphate | | |
| 13997 | Morphine Sulphate | | |
| 14050 | Morphine Sulphate | | |
| 14063 | Morphine sulphate | | |
| 14156 | Morphine sulphate | | |
| 14226 | Morphine sulphate | | |
| 14394 | Phenazocine Hydrobromide | | |
| 14900 | Fentanyl | | |
| 15064 | Morphine | | |
| 15337 | Fentanyl citrate | | |
| 15339 | Diamorphine Hydrochloride | | |
| 15350 | Fentanyl | | |
| 15353 | Papaveretum | | |
| 15514 | Diamorphine | | |
| 15781 | Morphine Sulphate | | |
| 15792 | Hydromorphone hydrochloride | | |
| 15793 | Diamorphine hydrochloride | | |
| 15798 | Hydromorphone hydrochloride | | |
| 15815 | Morphine sulphate | | |
| 15950 | Morphine sulphate | | |
| 15964 | Morphine sulphate | | |
| 16189 | Morphine Sulphate | | |
| 16273 | Morphine sulphate | | |
| 16335 | Morphine tartrate/Cyclizine tartrate | | |
| 16618 | Fentanyl | | |
| 16964 | Nalbuphine Hydrochloride | | |
| 17043 | Pethidine hydrochloride | | |
| 17092 | Morphine | | |
| 17163 | Diamorphine | | |
| 17167 | Phenazocine Hydrobromide | | |
| 17271 | Morphine sulphate | | |
| 17386 | Pethidine hydrochloride | | |
| 17398 | Morphine tartrate/Cyclizine tartrate | | |
| 17490 | Cyclizine Tartrate/Morphine Tartrate | | |
| 17734 | Dextromoramide Tartrate | | |
| 17825 | Morphine | | |
| 17863 | Pentazocine lactate | | |
| 17893 | Morphine sulphate | | |
| 17936 | Morphine sulphate | | |
| 17943 | Morphine sulphate | | |
| 18166 | Morphine Sulphate | | |
| 18174 | Fentanyl citrate | | |
| 18468 | Morphine anhydrous/Peppermint oil | | |
| 18491 | Pethidine hydrochloride/Promethazine hydrochloride | | |
| 18624 | Nepenthe Sol | | |
| 18626 | Morphine Sulphate | | |
| 18639 | Morphine Sulphate | | |
| 18656 | Morphine sulphate | | |
| 18700 | Morphine sulphate | | |
| 18727 | Morphine Sulphate | | |
| 18734 | Morphine sulphate | | |
| 18792 | Diamorphine hydrochloride | | |
| 18801 | Morphine sulphate | | |
| 18881 | Morphine sulphate | | |
| 18965 | Diamorphine | | |
| 18977 | Diamorphine hydrochloride | | |
| 19092 | Morphine Sulphate | | |
| 19116 | Pethidine hydrochloride/Promethazine hydrochloride | | |
| 19119 | Nalbuphine Hydrochloride | | |
| 19291 | Morphine sulphate | | |
| 19317 | Codeine Phosphate/Papaverine Hydrochloride/Morphine Hydrochloride | | |
| 19351 | Diamorphine | | |
| 19449 | Morphine sulphate | | |
| 19471 | Morphine sulphate | | |
| 19477 | Morphine sulphate | | |
| 19764 | Codeine Phosphate/Papaverine Hydrochloride/Morphine Hydrochloride | | |
| 19954 | Hydromorphone hydrochloride | | |
| 19972 | Hydromorphone hydrochloride | | |
| 20005 | Morphine Sulphate | | |
| 20008 | Morphine Sulphate | | |
| 20039 | Levorphanol Tartrate | | |
| 20219 | Morphine Sulphate | | |
| 20713 | Diamorphine | | |
| 20752 | Diamorphine | | |
| 20783 | Morphine sulphate | | |
| 20815 | Morphine sulphate | | |
| 21275 | Hydromorphone hydrochloride | | |
| 21285 | Hydromorphone hydrochloride | | |
| 21868 | Cocaine/Diamorphine Hydrochloride | | |
| 21972 | Morphine | | |
| 22024 | Morphine sulphate | | |
| 22026 | Morphine sulphate | | |
| 22066 | Fentanyl | | |
| 22571 | Morphine Sulphate/Atropine Sulphate | | |
| 22622 | Morphine/Cyclizine | | |
| 22690 | Morphine Sulphate | | |
| 22756 | Morphine sulphate | | |
| 22896 | Pethidine | | |
| 23060 | Morphine sulphate | | |
| 23063 | Morphine anhydrous/Peppermint oil | | |
| 23128 | Ammonium Chlor/Morphine | | |
| 23375 | Dextromoramide Tartrate | | |
| 23442 | Pethidine hydrochloride | | |
| 23775 | Morphine Sulphate | | |
| 23777 | Morphine Sulphate | | |
| 23778 | Diamorphine | | |
| 23785 | Diamorphine | | |
| 23906 | Alfentanil hydrochloride | | |
| 24108 | Diamorphine Hydrochloride | | |
| 24192 | Temgesic | | |
| 24414 | Temgesic | | |
| 24453 | Morphine Sulphate | | |
| 24640 | Diamorphine Hydrochloride | | |
| 24697 | Diamorphine | | |
| 24736 | Hydromorphone hydrochloride | | |
| 24790 | Alfentanil Hydrochloride | | |
| 24808 | Morphine Sulphate | | |
| 24816 | Morphine | | |
| 24830 | Morphine Sulphate | | |
| 24840 | Diamorphine | | |
| 24867 | Pethidine Hydrochloride/levallorphan | | |
| 24986 | Fentanyl citrate | | |
| 25185 | Fentanyl Citrate | | |
| 25199 | Fentanyl citrate | | |
| 25316 | Cyclizine Tartrate/Morphine Tartrate | | |
| 25481 | Morphine Anhydrous | | |
| 25503 | Ammonium Chloride/Morphine | | |
| 25611 | Dextromoramide Tartrate | | |
| 25649 | Diamorphine | | |
| 25650 | Morphine Sulphate | | |
| 25830 | Diamorphine | | |
| 25833 | Pentazocine lactate | | |
| 26021 | Fentanyl citrate | | |
| 26115 | Papaveretum/Hyoscine hydrobromide | | |
| 26283 | Morphine sulphate | | |
| 26284 | Morphine sulphate | | |
| 26407 | Diamorphine | | |
| 26805 | Morphine Sulphate | | |
| 26908 | Fentanyl citrate | | |
| 27058 | Morphine sulphate | | |
| 27298 | Morphine Sulphate | | |
| 27352 | Diamorphine | | |
| 27436 | Morphine Sulphate | | |
| 27548 | Oxycodone | | |
| 27749 | Morphine Sulphate | | |
| 28189 | Fentanyl | | |
| 28396 | Diamorphine | | |
| 28421 | Morphine Sulphate | | |
| 28503 | Morphine Sulphate | | |
| 28711 | Diamorphine Hydrochloride | | |
| 28732 | Hyoscine Hydrobromide/Papaveretum | | |
| 28805 | Dextromoramide Tartrate | | |
| 28837 | Morphine Sulphate | | |
| 29014 | Diamorphine Hydrochloride | | |
| 29020 | Morphine sulphate | | |
| 29379 | Morphine | | |
| 29426 | Pethidine Hydrochloride | | |
| 29500 | Diamorphine hydrochloride | | |
| 29577 | Fentanyl citrate | | |
| 29591 | Diamorphine | | |
| 29592 | Diamorphine | | |
| 29898 | Morphine sulphate | | |
| 30049 | Morphine Sulphate | | |
| 30252 | Morphine Anhydrous | | |
| 30320 | Diamorphine | | |
| 30514 | Diamorphine Hydrochloride | | |
| 30633 | Levorphanol Tartrate | | |
| 30698 | Diamorphine Hydrochloride | | |
| 30761 | Diamorphine Hydrochloride | | |
| 31033 | Diamorphine Hydrochloride | | |
| 31044 | Morphine Sulphate | | |
| 31053 | Fentanyl | | |
| 31253 | Pethidine Hydrochloride | | |
| 31407 | Ipecacuanha & Morphine Conc 1-4 Mix | | |
| 31582 | Pentazocine lactate | | |
| 31584 | Pentazocine lactate | | |
| 31599 | Morphine Sulphate | | |
| 31650 | Morphine Sulphate | | |
| 31885 | Pethidine hydrochloride | | |
| 31935 | Pethidine hydrochloride | | |
| 31960 | Diamorphine hydrochloride | | |
| 32357 | Morphine Sulphate | | |
| 32381 | Alfentanil hydrochloride | | |
| 32425 | Oxycodone hydrochloride | | |
| 32459 | Morphine Sulphate | | |
| 32460 | Morphine Sulphate | | |
| 32520 | Morphine anhydrous | | |
| 32687 | Diamorphine & Terpoin Lin | | |
| 32688 | Diamorphine Hydrochloride | | |
| 32831 | Pethidine Hydrochloride | | |
| 32897 | Diamorphine hydrochloride | | |
| 33954 | Pethidine Hydrochloride/Promethazine Hydrochloride | | |
| 34477 | Morphine sulphate | | |
| 34489 | Diamorphine Hydrochloride | | |
| 34771 | Morphine Sulphate | | |
| 34786 | Diamorphine hydrochloride | | |
| 34787 | Diamorphine hydrochloride | | |
| 35038 | Oxycodone hydrochloride | | |
| 35085 | Oxycodone hydrochloride | | |
| 35093 | Morphine Sulphate | | |
| 35169 | Buprenorphine Hydrochloride/naloxone Hydrochloride | | |
| 35170 | Buprenorphine hydrochloride/Naloxone hydrochloride | | |
| 35269 | Buprenorphine hydrochloride | | |
| 35330 | Fentanyl citrate | | |
| 35341 | Oxycodone hydrochloride | | |
| 35500 | Alfentanil hydrochloride | | |
| 35681 | Buprenorphine hydrochloride/Naloxone hydrochloride | | |
| 35682 | Buprenorphine Hydrochloride/naloxone Hydrochloride | | |
| 35853 | Fentanyl citrate | | |
| 35968 | Fentanyl | | |
| 36040 | Fentanyl | | |
| 36185 | Alfentanil hydrochloride | | |
| 36211 | Fentanyl | | |
| 37251 | Alfentanil hydrochloride | | |
| 37703 | Pethidine hydrochloride | | |
| 37719 | Fentanyl | | |
| 37779 | Fentanyl | | |
| 37923 | Fentanyl | | |
| 37928 | Fentanyl | | |
| 37954 | Fentanyl | | |
| 37960 | Fentanyl | | |
| 37968 | Fentanyl | | |
| 38013 | Pethidine hydrochloride | | |
| 38031 | Fentanyl | | |
| 38092 | Pentazocine Lactate | | |
| 38103 | Pethidine Hydrochloride | | |
| 38301 | Dipipanone Hydrochloride/Cyclizine Hydrochloride | | |
| 38311 | Buprenorphine Hydrochloride/Naloxone Hydrochloride | | |
| 38326 | Fentanyl | | |
| 38351 | Fentanyl | | |
| 38365 | Fentanyl | | |
| 38524 | Alfentanil hydrochloride | | |
| 38553 | Fentanyl | | |
| 39084 | Fentanyl | | |
| 39180 | Fentanyl | | |
| 39251 | Fentanyl | | |
| 39419 | Dextromoramide tartrate | | |
| 39469 | Fentanyl citrate | | |
| 39475 | Naloxone Hydrochloride/oxycodone Hydrochloride | | |
| 39477 | Naloxone Hydrochloride/oxycodone Hydrochloride | | |
| 39478 | Naloxone hydrochloride/Oxycodone hydrochloride | | |
| 39498 | Naloxone hydrochloride/Oxycodone hydrochloride | | |
| 39518 | Fentanyl citrate | | |
| 39590 | Fentanyl citrate | | |
| 39723 | Fentanyl Citrate | | |
| 39746 | Fentanyl citrate | | |
| 39756 | Fentanyl citrate | | |
| 39799 | Fentanyl citrate | | |
| 39842 | Meptazinol hydrochloride | | |
| 39929 | Fentanyl citrate | | |
| 39987 | Fentanyl citrate | | |
| 40018 | Fentanyl citrate | | |
| 40098 | Fentanyl Citrate | | |
| 40128 | Fentanyl Citrate | | |
| 40211 | Buprenorphine hydrochloride | | |
| 40212 | Buprenorphine hydrochloride | | |
| 40239 | Pethidine hydrochloride | | |
| 40427 | Alfentanil Hydrochloride | | |
| 40434 | Fentanyl citrate | | |
| 40473 | Buprenorphine hydrochloride | | |
| 40508 | Fentanyl citrate | | |
| 40563 | Morphine sulphate | | |
| 40576 | Fentanyl Citrate | | |
| 40616 | Naloxone Hydrochloride/oxycodone Hydrochloride | | |
| 40645 | Naloxone Hydrochloride/oxycodone Hydrochloride | | |
| 40688 | Oxycodone Hydrochloride | | |
| 40752 | Oxycodone hydrochloride | | |
| 40785 | Oxycodone hydrochloride/Naloxone hydrochloride | | |
| 40940 | Fentanyl Citrate | | |
| 40957 | Fentanyl citrate | | |
| 40961 | Oxycodone hydrochloride/Naloxone hydrochloride | | |
| 41135 | Fentanyl citrate | | |
| 41161 | Fentanyl | | |
| 41286 | Fentanyl Citrate | | |
| 41348 | Fentanyl Citrate | | |
| 41550 | Pethidine Hydrochloride | | |
| 41668 | Morphine hydrochloride | | |
| 41673 | Morphine sulphate | | |
| 41674 | Morphine sulphate | | |
| 41722 | Diamorphine hydrochloride | | |
| 41974 | Morphine sulphate | | |
| 42021 | Fentanyl | | |
| 42074 | Buprenorphine Hydrochloride/Naloxone Hydrochloride | | |
| 42094 | Pentazocine lactate | | |
| 42380 | Morphine Sulphate | | |
| 42399 | Fentanyl citrate | | |
| 42538 | Fentanyl citrate | | |
| 42576 | Fentanyl | | |
| 42590 | Fentanyl | | |
| 42591 | Fentanyl | | |
| 42708 | Pethidine Hydrochloride | | |
| 42913 | Diamorphine hydrochloride | | |
| 43089 | Fentanyl Citrate | | |
| 43152 | Fentanyl | | |
| 43315 | Morphine sulphate | | |
| 43617 | Fentanyl Citrate | | |
| 43652 | Morphine sulphate | | |
| 43657 | Morphine sulphate | | |
| 43720 | Alfentanil hydrochloride | | |
| 43812 | Fentanyl citrate | | |
| 44487 | Fentanyl | | |
| 44837 | Fentanyl | | |
| 44867 | Pentazocine lactate | | |
| 45092 | Fentanyl Citrate | | |
| 45325 | Pethidine Hydrochloride | | |
| 45439 | Fentanyl Citrate | | |
| 45460 | Fentanyl | | |
| 45549 | Fentanyl | | |
| 45598 | Fentanyl citrate | | |
| 45736 | Morphine sulphate | | |
| 45745 | Oxycodone hydrochloride | | |
| 45766 | Oxycodone hydrochloride | | |
| 45788 | Oxycodone hydrochloride | | |
| 45790 | Oxycodone hydrochloride | | |
| 45800 | Tapentadol hydrochloride | | |
| 45811 | Tapentadol hydrochloride | | |
| 45827 | Oxycodone hydrochloride | | |
| 45830 | Oxycodone hydrochloride | | |
| 45894 | Fentanyl Citrate | | |
| 45929 | Oxycodone hydrochloride | | |
| 45936 | Tapentadol hydrochloride | | |
| 45982 | Tapentadol hydrochloride | | |
| 46018 | Tapentadol Hydrochloride | | |
| 46019 | Tapentadol Hydrochloride | | |
| 46020 | Tapentadol Hydrochloride | | |
| 46021 | Tapentadol Hydrochloride | | |
| 46022 | Tapentadol hydrochloride | | |
| 46159 | Tapentadol Hydrochloride | | |
| 46187 | Oxycodone Hydrochloride | | |
| 46354 | Fentanyl citrate | | |
| 46461 | Tapentadol hydrochloride | | |
| 46555 | Fentanyl citrate | | |
| 46559 | Fentanyl | | |
| 46560 | Fentanyl | | |
| 46657 | Fentanyl | | |
| 46658 | Fentanyl | | |
| 46659 | Tapentadol hydrochloride | | |
| 46733 | Fentanyl | | |
| 47072 | Papaveretum | | |
| 47154 | Morphine sulphate | | |
| 47399 | Tapentadol Hydrochloride | | |
| 47413 | Fentanyl | | |
| 47460 | Tapentadol hydrochloride | | |
| 47555 | Morphine sulphate | | |
| 47671 | Diamorphine Hydrochloride | | |
| 47672 | Diamorphine Hydrochloride | | |
| 47753 | Morphine sulphate | | |
| 47759 | Fentanyl Citrate | | |
| 47867 | Morphine sulphate | | |
| 47949 | Morphine sulphate | | |
| 47985 | Morphine sulphate | | |
| 48128 | Pethidine hydrochloride | | |
| 48148 | Pethidine hydrochloride | | |
| 48158 | Diamorphine hydrochloride | | |
| 48183 | Fentanyl citrate | | |
| 48259 | Diamorphine hydrochloride | | |
| 48413 | Diamorphine hydrochloride | | |
| 48434 | Diamorphine hydrochloride | | |
| 48483 | Diamorphine hydrochloride | | |
| 48571 | Fentanyl | | |
| 48604 | Morphine sulphate | | |
| 48880 | Diamorphine hydrochloride | | |
| 48912 | Diamorphine hydrochloride | | |
| 48913 | Diamorphine hydrochloride | | |
| 48953 | Diamorphine hydrochloride | | |
| 49742 | Oxycodone hydrochloride | | |
| 49787 | Oxycodone hydrochloride | | |
| 49791 | Oxycodone hydrochloride | | |
| 49940 | Oxycodone hydrochloride | | |
| 49976 | Morphine sulphate | | |
| 50095 | Oxycodone hydrochloride | | |
| 50380 | Buprenorphine hydrochloride | | |
| 50513 | Morphine sulphate | | |
| 50671 | Fentanyl | | |
| 50726 | Oxycodone hydrochloride | | |
| 50733 | Oxycodone hydrochloride | | |
| 50929 | Fentanyl | | |
| 51235 | Fentanyl | | |
| 51384 | Oxycodone hydrochloride | | |
| 51611 | Oxycodone hydrochloride | | |
| 51789 | Oxycodone hydrochloride | | |
| 51896 | Oxycodone hydrochloride | | |
| 52178 | Fentanyl citrate | | |
| 52216 | Oxycodone hydrochloride | | |
| 52217 | Oxycodone hydrochloride | | |
| 52220 | Oxycodone hydrochloride | | |
| 52400 | Pethidine hydrochloride | | |
| 52592 | Oxycodone hydrochloride | | |
| 52809 | Oxycodone hydrochloride | | |
| 53062 | Hydromorphone hydrochloride | | |
| 53106 | Morphine sulphate | | |
| 53113 | Oxycodone hydrochloride | | |
| 53116 | Oxycodone hydrochloride | | |
| 53181 | Diamorphine hydrochloride | | |
| 53273 | Morphine | | |
| 53417 | Diamorphine hydrochloride | | |
| 53639 | Morphine sulphate | | |
| 53709 | Pethidine Hydrochloride | | |
| 53918 | Morphine sulphate | | |
| 53929 | Pethidine hydrochloride | | |
| 54017 | Morphine Sulphate | | |
| 54085 | Pethidine Hydrochloride | | |
| 54406 | Morphine sulphate | | |
| 54520 | Morphine Sulphate | | |
| 54694 | Oxycodone hydrochloride | | |
| 54790 | Pethidine hydrochloride | | |
| 54806 | Buprenorphine | | |
| 54979 | Fentanyl | | |
| 55052 | Morphine sulphate | | |
| 55206 | Morphine sulphate | | |
| 55221 | Diamorphine hydrochloride | | |
| 55304 | Nalbuphine hydrochloride | | |
| 55365 | Morphine | | |
| 55724 | Diamorphine | | |
| 55752 | Fentanyl | | |
| 55832 | Morphine sulphate | | |
| 55839 | Pethidine Hydrochloride | | |
| 55852 | Pethidine Hydrochloride | | |
| 56022 | Pethidine hydrochloride | | |
| 56202 | Morphine Sulphate | | |
| 56329 | Morphine Sulphate | | |
| 56544 | Morphine Sulphate | | |
| 56581 | Alfentanil Hydrochloride | | |
| 56665 | Oxycodone hydrochloride | | |
| 56670 | Fentanyl | | |
| 56671 | Buprenorphine | | |
| 56788 | Morphine sulphate | | |
| 57027 | Pethidine hydrochloride | | |
| 57033 | Oxycodone hydrochloride | | |
| 57052 | Oxycodone hydrochloride | | |
| 57454 | Buprenorphine hydrochloride | | |
| 57623 | Morphine sulphate | | |
| 57750 | Morphine sulphate | | |
| 58039 | Oxycodone hydrochloride | | |
| 58114 | Oxycodone hydrochloride | | |
| 58190 | Pethidine hydrochloride | | |
| 58217 | Oxycodone hydrochloride | | |
| 58273 | Buprenorphine hydrochloride | | |
| 58279 | Diamorphine hydrochloride | | |
| 58290 | Morphine sulphate | | |
| 58493 | Oxycodone hydrochloride | | |
| 58499 | Diamorphine hydrochloride | | |
| 58710 | Morphine sulphate | | |
| 58737 | Pethidine hydrochloride | | |
| 58766 | Buprenorphine | | |
| 58836 | Morphine sulphate | | |
| 58853 | Oxycodone hydrochloride | | |
| 58879 | Morphine | | |
| 59057 | Fentanyl citrate | | |
| 59146 | Buprenorphine | | |
| 59392 | Buprenorphine | | |
| 59443 | Fentanyl citrate | | |
| 59473 | Buprenorphine | | |
| 59482 | Fentanyl | | |
| 59490 | Fentanyl | | |
| 59584 | Morphine sulphate | | |
| 59618 | Buprenorphine | | |
| 59678 | Fentanyl citrate | | |
| 59865 | Oxycodone hydrochloride | | |
| 59970 | Buprenorphine hydrochloride | | |
| 60053 | Buprenorphine hydrochloride | | |
| 60080 | Alfentanil hydrochloride | | |
| 60082 | Morphine Sulphate | | |
| 60146 | Oxycodone hydrochloride | | |
| 60158 | Oxycodone hydrochloride | | |
| 60170 | Buprenorphine | | |
| 60196 | Oxycodone hydrochloride | | |
| 60477 | Fentanyl | | |
| 60507 | Morphine | | |
| 60518 | Morphine Sulphate | | |
| 60721 | Diamorphine hydrochloride | | |
| 60759 | Tapentadol hydrochloride | | |
| 60766 | Fentanyl | | |
| 60943 | Buprenorphine | | |
| 60950 | Morphine sulphate | | |
| 61086 | Fentanyl | | |
| 61100 | Buprenorphine hydrochloride | | |
| 61156 | Fentanyl | | |
| 61241 | Morphine sulphate | | |
| 61305 | Fentanyl | | |
| 61400 | Morphine sulphate | | |
| 61423 | Morphine sulphate | | |
| 61506 | Morphine sulphate | | |
| 61584 | Morphine sulphate | | |
| 61708 | Fentanyl citrate | | |
| 61744 | Morphine sulphate | | |
| 61764 | Tapentadol hydrochloride | | |
| 61779 | Oxycodone hydrochloride | | |
| 61836 | Oxycodone hydrochloride | | |
| 61918 | Morphine sulphate | | |
| 61935 | Oxycodone hydrochloride | | |
| 61936 | Oxycodone hydrochloride | | |
| 61942 | Morphine sulphate | | |
| 62322 | Oxycodone hydrochloride | | |
| 62675 | Buprenorphine hydrochloride | | |
| 62689 | Morphine hydrochloride | | |
| 62708 | Methadone hydrochloride | | |
| 62776 | Buprenorphine hydrochloride | | |
| 62874 | Buprenorphine hydrochloride | | |
| 62969 | Buprenorphine hydrochloride | | |
| 63139 | Fentanyl | | |
| 63182 | Pethidine hydrochloride | | |
| 63198 | Oxycodone hydrochloride | | |
| 63332 | Oxycodone hydrochloride | | |
| 63340 | Fentanyl | | |
| 63398 | Fentanyl citrate | | |
| 63423 | Morphine sulphate | | |
| 63593 | Morphine sulphate | | |
| 63640 | Buprenorphine hydrochloride | | |
| 63714 | Oxycodone hydrochloride | | |
| 63788 | Buprenorphine hydrochloride | | |
| 64150 | Oxycodone hydrochloride | | |
| 64155 | Buprenorphine hydrochloride | | |
| 64164 | Oxycodone hydrochloride | | |
| 64333 | Oxycodone hydrochloride | | |
| 64417 | Morphine sulphate | | |
| 64426 | Oxycodone hydrochloride | | |
| 64552 | Oxycodone hydrochloride | | |
| 64780 | Morphine sulphate | | |
| 64781 | Morphine sulphate | | |
| 64807 | Oxycodone hydrochloride | | |
| 64847 | Buprenorphine hydrochloride | | |
| 64860 | Morphine sulphate | | |
| 64965 | Oxycodone hydrochloride | | |
| 65157 | Buprenorphine hydrochloride | | |
| 65168 | Fentanyl | | |
| 65359 | Fentanyl | | |
| 65372 | Diamorphine hydrochloride | | |
| 65390 | Oxycodone hydrochloride | | |
| 65392 | Oxycodone hydrochloride | | |
| 65437 | Fentanyl | | |
| 65646 | Fentanyl citrate | | |
| 65932 | Oxycodone hydrochloride | | |
| 65933 | Oxycodone hydrochloride | | |
| 66280 | Buprenorphine | | |
| 66298 | Oxycodone hydrochloride | | |
| 66336 | Morphine Sulphate | | |
| 66463 | Buprenorphine | | |
| 66470 | Buprenorphine | | |
| 66606 | Oxycodone hydrochloride | | |
| 66616 | Oxycodone hydrochloride | | |
| 66619 | Oxycodone hydrochloride | | |
| 66654 | Diamorphine hydrochloride | | |
| 66689 | Buprenorphine | | |
| 66695 | Buprenorphine | | |
| 66760 | Oxycodone hydrochloride | | |
| 66815 | Morphine sulphate | | |
| 66837 | Oxycodone hydrochloride | | |
| 67018 | Buprenorphine | | |
| 67258 | Fentanyl | | |
| 67356 | Buprenorphine | | |
| 67425 | Fentanyl | | |
| 67446 | Oxycodone hydrochloride | | |
| 67474 | Fentanyl | | |
| 67766 | Fentanyl | | |
| 67830 | Fentanyl | | |
| 67901 | Buprenorphine | | |
| 68167 | Buprenorphine | | |
| 68172 | Buprenorphine | | |
| 68196 | Buprenorphine | | |
| 68209 | Fentanyl | | |
| 68241 | Buprenorphine | | |
| 68402 | Buprenorphine | | |
| 68472 | Buprenorphine | | |
| 68479 | Buprenorphine | | |
| 68559 | Buprenorphine | | |
| 68712 | Morphine hydrochloride | | |
| 68743 | Buprenorphine | | |
| 68797 | Oxycodone hydrochloride | | |
| 68848 | Buprenorphine | | |
| 68888 | Buprenorphine | | |
| 68889 | Buprenorphine | | |
| 68890 | Buprenorphine | | |
| 68910 | Naloxone hydrochloride dihydrate/Buprenorphine hydrochloride | | |
| 68988 | Buprenorphine hydrochloride | | |
| 68989 | Buprenorphine hydrochloride | | |
| 69023 | Fentanyl | | |
| 69243 | Buprenorphine | | |
| 69254 | Buprenorphine | | |
| 69315 | Buprenorphine | | |
| 69474 | Oxycodone hydrochloride | | |
| 69559 | Oxycodone hydrochloride | | |
| 69795 | Buprenorphine | | |
| 69942 | Buprenorphine hydrochloride | | |
| 69993 | Oxycodone hydrochloride | | |
| 70065 | Buprenorphine hydrochloride | | |
| 70066 | Buprenorphine hydrochloride | | |
| 70117 | Buprenorphine | | |
| 70124 | Buprenorphine | | |
| 70139 | Buprenorphine | | |
| 70283 | Naloxone hydrochloride dihydrate/Buprenorphine hydrochloride | | |
| 70376 | Fentanyl | | |
| 70460 | Buprenorphine | | |
| 70461 | Buprenorphine | | |
| 70464 | Buprenorphine hydrochloride | | |
| 70631 | Buprenorphine | | |
| 70810 | Fentanyl | | |
| 70988 | Fentanyl | | |
| 71170 | Pentazocine hydrochloride | | |
| 71310 | Buprenorphine | | |
| 71335 | Oxycodone hydrochloride | | |
| 71410 | Naloxone hydrochloride dihydrate/Buprenorphine hydrochloride | | |
| 71630 | Buprenorphine hydrochloride | | |
| 71695 | Buprenorphine hydrochloride | | |
| 71711 | Buprenorphine | | |
| 72098 | Buprenorphine | | |
| 72160 | Buprenorphine | | |
| 72225 | Morphine sulphate | | |
| 72342 | Fentanyl | | |
| 72820 | Fentanyl | | |
| 73009 | Morphine sulphate | | |
| 73146 | Buprenorphine hydrochloride | | |
| 73333 | Fentanyl | | |
| 73406 | Buprenorphine | | |
| 73428 | Oxycodone hydrochloride | | |
| 73545 | Buprenorphine | | |
| 73649 | Fentanyl | | |
| 73664 | Fentanyl | | |
| 73841 | Oxycodone hydrochloride | | |
| 74294 | Oxycodone hydrochloride | | |
| 74337 | Buprenorphine | | |
| 74549 | Oxycodone hydrochloride | | |
| 74562 | Buprenorphine | | |
| 74779 | Fentanyl | | |
| 74780 | Naloxone hydrochloride/Oxycodone hydrochloride | | |
| 74863 | Buprenorphine hydrochloride | | |
| 74908 | Oxycodone hydrochloride | | |
| 74909 | Oxycodone hydrochloride | | |
| 74910 | Oxycodone hydrochloride | | |
| 75283 | Oxycodone hydrochloride | | |
| 75334 | Buprenorphine | | |
| 75357 | Oxycodone hydrochloride | | |
| 75369 | Oxycodone hydrochloride | | |
| 75443 | Fentanyl citrate | | |
| 75624 | Oxycodone hydrochloride | | |
| 75980 | Oxycodone hydrochloride | | |
| 76236 | Oxycodone hydrochloride | | |
| 76394 | Oxycodone hydrochloride | | |
| 76485 | Morphine sulphate | | |
| 76649 | Oxycodone hydrochloride | | |
| 76692 | Oxycodone hydrochloride | | |
| 76698 | Oxycodone hydrochloride | | |
| 76699 | Oxycodone hydrochloride | | |
| 76724 | Fentanyl | | |
| 76755 | Oxycodone hydrochloride | | |
| 76895 | Oxycodone hydrochloride | | |
| 77078 | Oxycodone hydrochloride | | |
| 77709 | Oxycodone hydrochloride | | |
| 77740 | Oxycodone hydrochloride | | |
| 77879 | Oxycodone hydrochloride | | |
| 78005 | Oxycodone hydrochloride | | |
| 78142 | Buprenorphine | | |
| 78223 | Pentazocine hydrochloride | | |
| 78234 | Morphine hydrochloride | | |
| 78444 | Fentanyl | | |
| 78477 | Buprenorphine | | |
| 78584 | Morphine sulphate | | |
| 78750 | Buprenorphine | | |
| 78845 | Buprenorphine | | |
| 78941 | Oxycodone hydrochloride | | |
| 78942 | Oxycodone hydrochloride | | |
| 79112 | Oxycodone hydrochloride | | |
| 79446 | Fentanyl | | |
| 79584 | Oxycodone hydrochloride | | |
| 79802 | Oxycodone hydrochloride | | |
| 79815 | Oxycodone hydrochloride | | |
| 80226 | Oxycodone hydrochloride | | |
| 80227 | Oxycodone hydrochloride | | |
| 80228 | Oxycodone hydrochloride | | |
| 80323 | Fentanyl | | |
| 80339 | Buprenorphine | | |
| 80433 | Buprenorphine hydrochloride | | |
| 80456 | Pentazocine hydrochloride | | |
| 80613 | Buprenorphine | | |
| 80632 | Oxycodone hydrochloride | | |
| 80991 | Fentanyl | | |
| 81243 | Fentanyl | | |
| 81260 | Fentanyl citrate | | |
| 81382 | Fentanyl | | |
| 81708 | Buprenorphine | | |
| 81721 | Buprenorphine | | |
| 81724 | Fentanyl citrate | | |
| 81734 | Buprenorphine | | |
| 81776 | Oxycodone hydrochloride | | |
| 81816 | Buprenorphine | | |
| 81901 | Fentanyl | | |
| 81902 | Fentanyl | | |
| 82062 | Oxycodone hydrochloride | | |
| 82160 | Buprenorphine | | |
| 82200 | Fentanyl | | |
| 82221 | Oxycodone hydrochloride | | |
| 82249 | Oxycodone hydrochloride | | |
| 82341 | Buprenorphine | | |
| 82359 | Fentanyl | | |
| 82368 | Fentanyl | | |
| 82370 | Fentanyl | | |
| 82422 | Buprenorphine | | |
| 82483 | Fentanyl | | |
| 82575 | Buprenorphine hydrochloride | | |
| 82874 | Morphine sulphate | | |
| 82928 | Oxycodone hydrochloride | | |

**Gabapentin Code List**

| **Prodcode** | **Drug Substance Name** |
| --- | --- |
| 660 | Gabapentin |
| 1584 | Gabapentin |
| 4781 | Gabapentin |
| 5221 | Gabapentin |
| 6304 | Gabapentin |
| 7538 | Gabapentin |
| 9979 | Gabapentin |
| 10007 | Gabapentin |
| 16215 | Gabapentin |
| 16404 | Gabapentin |
| 17564 | Gabapentin |
| 18211 | Gabapentin |
| 25815 | Gabapentin |
| 27454 | Gabapentin |
| 28713 | Gabapentin |
| 34506 | Gabapentin |
| 34606 | Gabapentin |
| 34716 | Gabapentin |
| 34946 | Gabapentin |
| 44022 | Gabapentin |
| 44187 | Gabapentin |
| 44261 | Gabapentin |
| 47579 | Gabapentin |
| 48035 | Gabapentin |
| 48060 | Gabapentin |
| 51118 | Gabapentin |
| 53296 | Gabapentin |
| 53784 | Gabapentin |
| 54609 | Gabapentin |
| 55008 | Gabapentin |
| 55535 | Gabapentin |
| 55624 | Gabapentin |
| 57120 | Gabapentin |
| 57527 | Gabapentin |
| 57649 | Gabapentin |
| 58162 | Gabapentin |
| 58382 | Gabapentin |
| 58383 | Gabapentin |
| 58472 | Gabapentin |
| 58960 | Gabapentin |
| 59147 | Gabapentin |
| 59196 | Gabapentin |
| 60389 | Gabapentin |
| 61266 | Gabapentin |
| 63375 | Gabapentin |
| 63432 | Gabapentin |
| 64213 | Gabapentin |
| 64302 | Gabapentin |
| 64306 | Gabapentin |
| 64981 | Gabapentin |
| 66617 | Gabapentin |
| 67091 | Gabapentin |
| 67969 | Gabapentin |
| 68047 | Gabapentin |
| 68049 | Gabapentin |
| 69914 | Gabapentin |
| 70247 | Gabapentin |
| 70459 | Gabapentin |
| 70506 | Gabapentin |
| 70738 | Gabapentin |
| 70954 | Gabapentin |
| 71013 | Gabapentin |
| 72849 | Gabapentin |
| 73047 | Gabapentin |
| 73587 | Gabapentin |
| 73635 | Gabapentin |
| 76014 | Gabapentin |
| 76415 | Gabapentin |
| 76435 | Gabapentin |
| 76604 | Gabapentin |
| 77136 | Gabapentin |
| 77695 | Gabapentin |
| 78410 | Gabapentin |
| 78641 | Gabapentin |
| 78801 | Gabapentin |
| 79400 | Gabapentin |
| 80385 | Gabapentin |
| 80504 | Gabapentin |
| 81124 | Gabapentin |
| 81635 | Gabapentin |
| 82381 | Gabapentin |
| 82447 | Gabapentin |
| 83011 | Gabapentin |
| 83012 | Gabapentin |
| 84405 | Gabapentin |
| 84749 | Gabapentin |
| 85437 | Gabapentin |

**Pregabalin Code List**

| **Prodcode** | | **Drug Substance Name** | |
| --- | --- | --- | --- |
| 790 | Pregabalin | |  |
| 819 | Pregabalin | |  |
| 6584 | Pregabalin | |  |
| 6631 | Pregabalin | |  |
| 6936 | Pregabalin | |  |
| 6949 | Pregabalin | |  |
| 6999 | Pregabalin | |  |
| 7005 | Pregabalin | |  |
| 7208 | Pregabalin | |  |
| 7209 | Pregabalin | |  |
| 7394 | Pregabalin | |  |
| 10189 | Pregabalin | |  |
| 16509 | Pregabalin | |  |
| 16542 | Pregabalin | |  |
| 37801 | Pregabalin | |  |
| 38293 | Pregabalin | |  |
| 48253 | Pregabalin | |  |
| 51227 | Pregabalin | |  |
| 51924 | Pregabalin | |  |
| 52547 | Pregabalin | |  |
| 55972 | Pregabalin | |  |
| 60543 | Pregabalin | |  |
| 63069 | Pregabalin | |  |
| 63088 | Pregabalin | |  |
| 63089 | Pregabalin | |  |
| 63090 | Pregabalin | |  |
| 63091 | Pregabalin | |  |
| 63174 | Pregabalin | |  |
| 63300 | Pregabalin | |  |
| 63317 | Pregabalin | |  |
| 63877 | Pregabalin | |  |
| 63964 | Pregabalin | |  |
| 63965 | Pregabalin | |  |
| 64005 | Pregabalin | |  |
| 64037 | Pregabalin | |  |
| 64038 | Pregabalin | |  |
| 64039 | Pregabalin | |  |
| 64040 | Pregabalin | |  |
| 64041 | Pregabalin | |  |
| 64042 | Pregabalin | |  |
| 64285 | Pregabalin | |  |
| 64497 | Pregabalin | |  |
| 64568 | Pregabalin | |  |
| 65069 | Pregabalin | |  |
| 65073 | Pregabalin | |  |
| 65218 | Pregabalin | |  |
| 65606 | Pregabalin | |  |
| 65787 | Pregabalin | |  |
| 65863 | Pregabalin | |  |
| 66509 | Pregabalin | |  |
| 66941 | Pregabalin | |  |
| 67053 | Pregabalin | |  |
| 67184 | Pregabalin | |  |
| 67384 | Pregabalin | |  |
| 67440 | Pregabalin | |  |
| 68014 | Pregabalin | |  |
| 68441 | Pregabalin | |  |
| 69034 | Pregabalin | |  |
| 69125 | Pregabalin | |  |
| 69296 | Pregabalin | |  |
| 69418 | Pregabalin | |  |
| 69497 | Pregabalin | |  |
| 69498 | Pregabalin | |  |
| 69499 | Pregabalin | |  |
| 69501 | Pregabalin | |  |
| 69554 | Pregabalin | |  |
| 69781 | Pregabalin | |  |
| 69799 | Pregabalin | |  |
| 69877 | Pregabalin | |  |
| 69987 | Pregabalin | |  |
| 70064 | Pregabalin | |  |
| 70229 | Pregabalin | |  |
| 70478 | Pregabalin | |  |
| 70544 | Pregabalin | |  |
| 70545 | Pregabalin | |  |
| 70546 | Pregabalin | |  |
| 70648 | Pregabalin | |  |
| 70729 | Pregabalin | |  |
| 70730 | Pregabalin | |  |
| 70731 | Pregabalin | |  |
| 70735 | Pregabalin | |  |
| 71221 | Pregabalin | |  |
| 71313 | Pregabalin | |  |
| 71461 | Pregabalin | |  |
| 71533 | Pregabalin | |  |
| 71659 | Pregabalin | |  |
| 72068 | Pregabalin | |  |
| 73026 | Pregabalin | |  |
| 73387 | Pregabalin | |  |
| 73424 | Pregabalin | |  |
| 73455 | Pregabalin | |  |
| 73584 | Pregabalin | |  |
| 73817 | Pregabalin | |  |
| 76318 | Pregabalin | |  |
| 77221 | Pregabalin | |  |
| 77847 | Pregabalin | |  |
| 78146 | Pregabalin | |  |
| 79320 | Pregabalin | |  |
| 80333 | Pregabalin | |  |
| 80564 | Pregabalin | |  |
| 80667 | Pregabalin | |  |
| 80705 | Pregabalin | |  |
| 80743 | Pregabalin | |  |
| 80762 | Pregabalin | |  |
| 80801 | Pregabalin | |  |
| 81501 | Pregabalin | |  |
| 81735 | Pregabalin | |  |
| 82071 | Pregabalin | |  |
| 82114 | Pregabalin | |  |
| 82261 | Pregabalin | |  |
| 82808 | Pregabalin | |  |
| 83073 | Pregabalin | |  |
| 83394 | Pregabalin | |  |
| 84457 | Pregabalin | |  |
| 85091 | Pregabalin | |  |

**Benzodiazepine Code List**

| **Prodcode** | **Drug Substance Name** |
| --- | --- |
| 20 | Temazepam |
| 35 | Nitrazepam |
| 46 | Diazepam |
| 47 | Diazepam |
| 664 | Lorazepam |
| 780 | Temazepam |
| 816 | Clonazepam |
| 921 | Temazepam |
| 1088 | Lorazepam |
| 1400 | Diazepam |
| 1463 | Chlordiazepoxide |
| 1559 | Clonazepam |
| 1729 | Temazepam |
| 2073 | Clonazepam |
| 2091 | Lorazepam |
| 2122 | Chlordiazepoxide |
| 2352 | Diazepam |
| 2401 | Valium |
| 2403 | Temazepam |
| 2404 | Triazolam |
| 2407 | Nitrazepam |
| 2737 | Rohypnol |
| 3105 | Dalmane |
| 3109 | Clobazam |
| 3110 | Clobazam |
| 3111 | Clobazam |
| 3147 | Chlordiazepoxide |
| 3205 | Diazepam |
| 3234 | Midazolam |
| 3250 | Tranxene |
| 3354 | Lormetazepam |
| 3357 | Lormetazepam |
| 3524 | Mogadon |
| 3686 | Nitrazepam |
| 3687 | Lormetazepam |
| 3870 | Diazepam |
| 3950 | Flurazepam |
| 3956 | Dalmane |
| 3973 | Tensium |
| 4140 | Oxazepam |
| 4141 | Oxazepam |
| 4338 | Valium |
| 4439 | Libraxin |
| 4483 | Clonazepam |
| 4543 | Chlordiazepoxide |
| 4566 | Oxazepam |
| 5150 | Loprazolam |
| 5294 | Chlordiazepoxide |
| 5856 | Hypnovel |
| 5872 | Midazolam |
| 6020 | Midazolam |
| 6025 | Chlordiazepoxide |
| 6516 | Chlordiazepoxide |
| 7301 | Midazolam |
| 7391 | Midazolam |
| 7392 | Midazolam |
| 7444 | Ativan |
| 7566 | Flurazepam |
| 7567 | Temazepam |
| 7569 | Temazepam |
| 7571 | Triazolam |
| 7652 | Oxazepam |
| 7786 | Mogadon |
| 7797 | Tranxene |
| 7924 | Nitrazepam |
| 8029 | Rivotril |
| 8487 | Frisium |
| 8550 | Chlordiazepoxide |
| 8721 | Oxazepam |
| 8742 | Clidinium |
| 8798 | Temazepam |
| 8913 | Librium |
| 9045 | Diazepam |
| 9048 | Librium |
| 9065 | Diazepam |
| 9111 | Diazepam |
| 9430 | Diazepam |
| 9696 | Alprazolam |
| 9721 | Lexotan |
| 9814 | Nitrazepam |
| 10274 | Diazepam |
| 10278 | Epistatus |
| 10402 | Valium |
| 10409 | Lorazepam |
| 10430 | Temazepam |
| 10581 | Hypnovel |
| 10650 | Rivotril |
| 10694 | Clobazam |
| 10789 | Nobrium |
| 10790 | Medazepam |
| 10791 | Nobrium |
| 10802 | Xanax |
| 10909 | Diazepam |
| 10954 | Ativan |
| 11486 | Alprazolam |
| 11958 | Lexotan |
| 11963 | Limbitrol |
| 12130 | Anxon |
| 12278 | Flurazepam |
| 12293 | Normison |
| 12452 | Anxon |
| 12462 | Temazepam |
| 12477 | Librium |
| 12598 | Xanax |
| 12849 | Diazepam |
| 13200 | Clonazepam |
| 13279 | Lorazepam |
| 13304 | Midazolam |
| 13305 | Midazolam |
| 13612 | Temazepam |
| 14388 | Frisium |
| 14417 | Lorazepam |
| 14425 | Halcion |
| 14480 | Flunitrazepam |
| 14534 | Limbitrol |
| 14743 | Clonazepam |
| 15110 | Temazepam |
| 15429 | Clobazam |
| 15492 | Nitrados |
| 16169 | Librium |
| 17038 | Rivotril |
| 17054 | Clobazam |
| 17294 | Librium |
| 17637 | Clonazepam |
| 17830 | Ativan |
| 17897 | Nitrados |
| 18125 | Librium |
| 18260 | Halcion |
| 18291 | Noctamid |
| 18342 | Amitriptyline |
| 18350 | Clobazam |
| 18928 | Flurazepam |
| 19299 | Valium |
| 19450 | Mogadon |
| 19941 | Bromazepam |
| 20164 | Valium |
| 20245 | Temazepam |
| 20692 | Chlordiazepoxide |
| 20701 | Chlordiazepoxide |
| 20801 | Temazepam |
| 20966 | Clobazam |
| 20968 | Diazepam |
| 21081 | Amitriptyline |
| 21437 | Loramet |
| 21454 | Normison |
| 21464 | Medazepam |
| 22189 | Chlordiazepoxide |
| 22424 | Bromazepam |
| 23002 | Lorazepam |
| 23120 | Temazepam |
| 23205 | Dormonoct |
| 23493 | Temazepam |
| 23726 | Noctamid |
| 23752 | Euhypnos |
| 23796 | Ketazolam |
| 23874 | Somnite |
| 24321 | Paxane |
| 24386 | Xanax |
| 24422 | Flurazepam |
| 24599 | Librium |
| 24739 | Temazepam |
| 24843 | Serenid |
| 25007 | Chlordiazepozide |
| 25273 | Oxanid |
| 25893 | Centrax |
| 26496 | Rivotril |
| 26835 | Midazolam |
| 26837 | Xanax |
| 26961 | Midazolam |
| 27367 | Temazepam |
| 27847 | Surem |
| 27880 | Tropium |
| 28058 | Temazepam |
| 28321 | Chlordiazepoxide |
| 28347 | Diazepam |
| 28360 | Ketazolam |
| 28703 | Evacalm |
| 28879 | Chlordiazepoxide |
| 29441 | Euhypnos |
| 29806 | Clobazam |
| 29945 | Diazepam |
| 30273 | Tropium |
| 30321 | Valium |
| 30779 | Temazepam |
| 30985 | Temazepam |
| 31163 | Temazepam |
| 32231 | Librium |
| 32296 | Diazepam |
| 32320 | Temazepam |
| 32500 | Clonazepam |
| 32847 | Temazepam |
| 32853 | Diazepam |
| 32911 | Midazolam |
| 33070 | Solis |
| 33086 | Lorazepam |
| 33648 | Temazepam |
| 33672 | Diazepam |
| 34002 | Temazepam |
| 34045 | Diazepam |
| 34292 | Lormetazepam |
| 34293 | Diazepam |
| 34331 | Temazepam |
| 34335 | Diazepam |
| 34338 | Diazepam |
| 34340 | Diazepam |
| 34361 | Lormetazepam |
| 34406 | Temazepam |
| 34408 | Nitrazepam |
| 34482 | Diazepam |
| 34491 | Clonazepam |
| 34508 | Temazepam |
| 34516 | Lormetazepam |
| 34524 | Diazepam |
| 34534 | Lormetazepam |
| 34555 | Nitrazepam |
| 34561 | Diazepam |
| 34572 | Temazepam |
| 34615 | Diazepam |
| 34635 | Diazepam |
| 34642 | Lormetazepam |
| 34677 | Diazepam |
| 34681 | Diazepam |
| 34686 | Nitrazepam |
| 34692 | Lormetazepam |
| 34735 | Midazolam |
| 34770 | Nitrazepam |
| 34806 | Nitrazepam |
| 34807 | Diazepam |
| 34876 | Diazepam |
| 34892 | Diazepam |
| 34928 | Chlordiazepoxide |
| 34964 | Nitrazepam |
| 35373 | Midazolam |
| 35932 | Lorazepam |
| 35936 | Chlordiazepoxide |
| 36200 | Lorazepam |
| 36581 | Atensine |
| 36602 | Temazepam |
| 36604 | Oxazepam |
| 36611 | Euhypnos |
| 37124 | Midazolam |
| 37325 | Remnos |
| 37566 | Lorazepam |
| 37696 | Clobazam |
| 37745 | Lorazepam |
| 38193 | Midazolam |
| 38418 | Temazepam |
| 38424 | Temazepam |
| 39284 | Lorazepam |
| 40386 | Chlordiazepoxide |
| 41303 | Clobazam |
| 41385 | Nitrazepam |
| 41391 | Lorazepam |
| 41411 | Oxazepam |
| 41516 | Temazepam |
| 41531 | Oxazepam |
| 41542 | Oxazepam |
| 41553 | Oxazepam |
| 41562 | Temazepam |
| 41574 | Chlordiazepoxide |
| 41581 | Chlordiazepoxide |
| 41582 | Chlordiazepoxide |
| 41583 | Chlordiazepoxide |
| 41596 | Loprazolam |
| 41601 | Oxazepam |
| 41602 | Oxazepam |
| 41606 | Chlordiazepoxide |
| 41607 | Diazepam |
| 41629 | Chlordiazepoxide |
| 41632 | Diazepam |
| 41653 | Temazepam |
| 41717 | Temazepam |
| 41718 | Temazepam |
| 41822 | Triazolam |
| 41900 | Nitrazepam |
| 41988 | Chlordiazepoxide |
| 42503 | Diazepam |
| 42814 | Lorazepam |
| 43438 | Chlordiazepoxide |
| 43450 | Midazolam |
| 44302 | Tropium |
| 44764 | Midazolam |
| 45077 | Clonazepam |
| 45135 | Diazepam |
| 45218 | Diazepam |
| 45241 | Chlordiazepoxide |
| 45244 | Diazepam |
| 45254 | Temazepam |
| 45283 | Temazepam |
| 45313 | Diazepam |
| 45615 | Midazolam |
| 45695 | Midazolam |
| 45829 | Lorazepam |
| 45974 | Buccolam |
| 46078 | Temazepam |
| 46667 | Midazolam |
| 46757 | Midazolam |
| 46796 | Clobazam |
| 46797 | Buccolam |
| 46826 | Buccolam |
| 46850 | Clobazam |
| 46883 | Midazolam |
| 46896 | Lorazepam |
| 46909 | Triazolam |
| 46913 | Diazepam |
| 46939 | Temazepam |
| 46946 | Oxazepam |
| 46953 | Nitrazepam |
| 46964 | Temazepam |
| 46966 | Diazepam |
| 47045 | Buccolam |
| 47066 | Clonazepam |
| 48010 | Clobazam |
| 48517 | Lormetazepam |
| 48544 | Clonazepam |
| 48715 | Midazolam |
| 48807 | Midazolam |
| 48817 | Midazolam |
| 48818 | Midazolam |
| 49095 | Midazolam |
| 49534 | Midazolam |
| 49589 | Temazepam |
| 49595 | Midazolam |
| 50108 | Clonazepam |
| 50389 | Clobazam |
| 50618 | Midazolam |
| 51335 | Diazepam |
| 51425 | Clobazam |
| 51550 | Epistatus |
| 51925 | Epistatus |
| 51985 | Diazepam |
| 52052 | Clobazam |
| 52056 | Clobazam |
| 52093 | Clobazam |
| 52680 | Clobazam |
| 52845 | Midazolam |
| 52954 | Midazolam |
| 53306 | Epistatus |
| 53311 | Clonazepam |
| 53461 | Diazepam |
| 53566 | Diazepam |
| 53739 | Clonazepam |
| 53748 | Midazolam |
| 54695 | Diazepam |
| 54759 | Clobazam |
| 54919 | Epistatus |
| 54934 | Tapclob |
| 55303 | Triazolam |
| 55481 | Clobazam |
| 55642 | Epistatus |
| 55774 | Bromazepam |
| 55836 | Temazepam |
| 55931 | Midazolam |
| 56236 | Diazepam |
| 56551 | Lorazepam |
| 56781 | Tapclob |
| 56811 | Temazepam |
| 56927 | Temazepam |
| 56988 | Midazolam |
| 57268 | Lorazepam |
| 57510 | Frisium |
| 57596 | Midazolam |
| 57664 | Clonazepam |
| 57749 | Diazepam |
| 57838 | Diazepam |
| 58460 | Clonazepam |
| 58482 | Clonazepam |
| 58685 | Midazolam |
| 58959 | Diazepam |
| 59122 | Diazepam |
| 59396 | Clonazepam |
| 59407 | Diazepam |
| 59913 | Midazolam |
| 60320 | Clobazam |
| 60825 | Temazepam |
| 60936 | Diazepam |
| 61015 | Clonazepam |
| 61072 | Midazolam |
| 61290 | Midazolam |
| 61450 | Lorazepam |
| 61626 | Clonazepam |
| 61678 | Nitrazepam |
| 61753 | Midazolam |
| 61833 | Midazolam |
| 61886 | Lorazepam |
| 62216 | Diazepam |
| 62270 | Chlordiazepoxide |
| 62402 | Midazolam |
| 62541 | Diazepam |
| 62645 | Temazepam |
| 62926 | Clobazam |
| 63238 | Diazepam |
| 63662 | Midazolam |
| 63665 | Noctamid |
| 63674 | Temazepam |
| 63686 | Midazolam |
| 63694 | Diazepam |
| 63985 | Midazolam |
| 64200 | Diazepam |
| 64470 | Chlordiazepoxide |
| 64505 | Clonazepam |
| 64693 | Clonazepam |
| 64729 | Lorazepam |
| 64775 | Nitrazepam |
| 64876 | Lorazepam |
| 64879 | Clobazam |
| 65135 | Nitrazepam |
| 65238 | Clonazepam |
| 66340 | Midazolam |
| 66745 | Loprazolam |
| 66878 | Buccolam |
| 66879 | Midazolam |
| 66889 | Buccolam |
| 66891 | Buccolam |
| 67007 | Buccolam |
| 67016 | Midazolam |
| 67031 | Midazolam |
| 67032 | Midazolam |
| 67192 | Diazepam |
| 67193 | Diazepam |
| 67297 | Diazepam |
| 67451 | Diazepam |
| 67554 | Lorazepam |
| 67570 | Midazolam |
| 67785 | Diazepam |
| 67957 | Diazepam |
| 68414 | Diazepam |
| 68423 | Midazolam |
| 68691 | Midazolam |
| 69000 | Midazolam |
| 69211 | Clonazepam |
| 69437 | Perizam |
| 69764 | Perizam |
| 69783 | Diazepam |
| 69810 | Diazepam |
| 69976 | Clobazam |
| 70045 | Midazolam |
| 70071 | Temazepam |
| 70255 | Clobazam |
| 70536 | Diazepam |
| 70561 | Rivotril |
| 70663 | Midazolam |
| 70682 | Lorazepam |
| 70981 | Clobazam |
| 71063 | Midazolam |
| 71073 | Nitrazepam |
| 71245 | Diazepam |
| 71336 | Diazepam |
| 71813 | Clobazam |
| 72550 | Rivotril |
| 72551 | Midazolam |
| 72552 | Midazolam |
| 72832 | Clobazam |
| 73423 | Midazolam |
| 73483 | Temazepam |
| 73526 | Diazepam |
| 73660 | Nitrazepam |
| 73661 | Clobazam |
| 73746 | Rivotril |
| 73751 | Diazepam |
| 73815 | Diazepam |
| 74065 | Chlordiazepoxide |
| 74219 | Clonazepam |
| 74342 | Diazepam |
| 74422 | Clonazepam |
| 74433 | Clonazepam |
| 74584 | Clonazepam |
| 74743 | Lorazepam |
| 74755 | Diazepam |
| 74783 | Temazepam |
| 74865 | Clonazepam |
| 74866 | Clonazepam |
| 74867 | Clonazepam |
| 74934 | Midazolam |
| 74984 | Diazepam |
| 75197 | Nitrazepam |
| 75201 | Midazolam |
| 75415 | Epistatus |
| 75473 | Diazepam |
| 75571 | Clonazepam |
| 75594 | Temazepam |
| 76192 | Diazepam |
| 76218 | Lorazepam |
| 76490 | Temazepam |
| 76592 | Clonazepam |
| 76757 | Clonazepam |
| 76758 | Midazolam |
| 77021 | Midazolam |
| 77022 | Midazolam |
| 77463 | Rivotril |
| 77536 | Atensine |
| 77589 | Nitrazepam |
| 77652 | Chlordiazepoxide |
| 77686 | Temazepam |
| 77799 | Zacco |
| 77999 | Clobazam |
| 78243 | Lorazepam |
| 78306 | Diazepam |
| 78312 | Lorazepam |
| 78323 | Lorazepam |
| 78362 | Chlordiazepoxide |
| 78434 | Lorazepam |
| 78738 | Diazepam |
| 79375 | Nitrazepam |
| 79387 | Chlordiazepoxide |
| 79472 | Midazolam |
| 79487 | Temazepam |
| 79687 | Midazolam |
|  |  |

**Z-Drug Code List**

| **Prodcode** | **Drug Substance Name** |
| --- | --- |
| 66 | Zopiclone |
| 721 | Zopiclone |
| 2017 | Zolpidem tartrate |
| 3126 | Zolpidem tartrate |
| 3320 | Zopiclone |
| 3741 | Zolpidem tartrate |
| 4187 | Zopiclone |
| 5058 | Zopiclone |
| 5306 | Zaleplon |
| 5352 | Zaleplon |
| 5459 | Zolpidem tartrate |
| 5916 | Zaleplon |
| 9598 | Zaleplon |
| 14365 | Zopiclone |
| 15852 | Zopiclone |
| 24135 | Zopiclone |
| 29219 | Zopiclone |
| 29869 | Zolpidem tartrate |
| 30056 | Zopiclone |
| 30377 | Zopiclone |
| 30981 | Zolpidem tartrate |
| 31710 | Zolpidem tartrate |
| 33045 | Zopiclone |
| 33663 | Zopiclone |
| 33841 | Zolpidem tartrate |
| 34372 | Zopiclone |
| 34612 | Zopiclone |
| 34777 | Zopiclone |
| 34823 | Zopiclone |
| 34874 | Zopiclone |
| 34897 | Zopiclone |
| 41539 | Zolpidem tartrate |
| 41696 | Zolpidem tartrate |
| 41697 | Zolpidem tartrate |
| 42089 | Zolpidem tartrate |
| 43445 | Zopiclone |
| 43560 | Zolpidem tartrate |
| 45353 | Zopiclone |
| 46799 | Zopiclone |
| 52022 | Zopiclone |
| 57937 | Zopiclone |
| 59640 | Zopiclone |
| 61477 | Zopiclone |
| 63592 | Zopiclone |
| 65190 | Zolpidem tartrate |
| 65637 | Zopiclone |
| 70727 | Zopiclone |
| 71089 | Zopiclone |
| 73154 | Zopiclone |
